# Site-Dependent Differences in DNA Methylation and Their Impact on Plant Establishment and Phosphorus Nutrition in *Populus trichocarpa*

**DOI:** 10.1371/journal.pone.0168623

**Published:** 2016-12-19

**Authors:** Brigitte Schönberger, Xiaochao Chen, Svenja Mager, Uwe Ludewig

**Affiliations:** Crop Science Institute, Department of Nutritional Crop Physiology, University of Hohenheim, Stuttgart, Germany; Universität Stuttgart, GERMANY

## Abstract

The propagation via clonal stem cuttings is a frequent practice in tree plantations. Despite their clonal origin, the trees establish differently according to weather, temperature and nutrient availability, as well as the presence of various stresses. Here, clonal *Populus trichocarpa* (cv. Muhle Larson) cuttings from different sites were transferred into a common, fully nutrient supplied environment. Despite identical underlying genetics, stem cuttings derived from sites with lower phosphorus availability established worse, independent of phosphorus (P) level after transplantation. Differential growth of material from the sites was reflected in differences in the whole genome DNA methylome. Methylation differences were sequence context-dependent, but differentially methylated regions (DMRs) were apparently unrelated to P nutrition genes. Despite the undisputed negative general correlation of DNA promoter methylation with gene repression, only few of the top-ranked DMRs resulted in differential gene expression in roots or shoots. However, differential methylation was associated with site-dependent, different total amounts of microRNAs (miRNAs), with few miRNAs sequences directly targeted by differential methylation. Interestingly, in roots and shoots, the miRNA amount was dependent on the previous habitat and changed in roots in a habitat-dependent way under phosphate starvation conditions. Differentially methylated miRNAs, together with their target genes, showed P-dependent expression profiles, indicating miRNA expression differences as a P-related epigenetic modification in poplar. Together with differences in DNA methylation, such epigenetic mechanisms may explain habitat or seasonal memory in perennials and site-dependent growth performances.

## Introduction

Epigenetics describes the heritable epigenetic information without any changes in nucleotide sequence [1]. Besides histone modifications and RNA interference, DNA methylation is considered as a classic epigenetic mechanism leading to heritable differences in gene expression, especially by methylation of transposable elements [2]. In addition, there is strong evidence that DNA methylation in promoter regions silences genes, whereas DNA methylation in gene body sequences is positively correlated to gene expression in mammals [3] and in different plant species like *Arabidopsis* [4], rice [5] and poplar [6]. Nevertheless, most of these correlations exist on a whole methylome/transcriptome scale, but whether causal relationships between gene expression and DNA methylation exist, is poorly understood.

Another important aspect of differential DNA methylation is the cytosine (C) context, in which the methylation occurs (CpG, CHG and CHH, where H represents A, T or C). In plants, methylations are observed in both symmetrical (CpG and CHG) and asymmetrical (CHH) sequences [7]. CpG methylations are maintained via the methyltransferase 1 (MET1) and therefore easily transmitted through cycles of DNA replication, via mitotic and meiotic divisions [8,9]. Methylation in the CpG context is considered as inheritable or conserved methylation, especially in heterochromatic regions of the chromosome. By contrast, chromomethylase 3 (CMT3) and domains rearranged methylase (DRM) genes are responsible for so called “*de novo*” methylations appearing mainly in CHG and CHH contexts. These can be set during adaptive stress responses [10,11], while the heritability of adaptive traits is questioned, because of strong epigenetic resetting in the germline. However, CHG methylations are also symmetrical sequences that are easily transferred during cell replication and thus can be inherited as well. Because in perennial plants, such as poplar, annual resetting via seeds does not occur, epigenetic adaptive stress responses might have an additional role in seasonal or site-specific memory. This has practical consequences, as in short rotation forestry, clonal and vegetatively propagated material is used and “*de novo*” methylations might transfer seasonal (annual) memory due to a lack of genetic recombination via seed propagation to “inherit” adaptive traits [12–14].

Alongside already well-investigated mammalian epigenetics [15–17], recent studies in plants have recently uncovered a major role of epigenetic adaptation in gene expression alteration due to environmental stress [18,19]. Most notably, Phosphorus (P) availability seems to transiently trigger epigenetic modifications, e.g. Phosphate (P_i_) starvation and recovery conditions induce methylation in transposable elements close to highly induced P-related genes in rice [5]. Similar P_i_ starvation induced responses were also found in *Arabidopsis* [5], but in this species the P_i_ starvation response was described to be regulated by chromatin remodeling of H2A.Z histones, which are anti-correlated with DNA methylation [20].

Besides DNA methylation, miRNAs contain extra-chromosomal sequence-specific information and are also involved in epigenetic mechanisms. They are small (~ 21–24 nucleotides), non-coding, single-stranded RNAs and a subset of these act as key regulators of gene expression, mainly by transcriptional or post-transcriptional silencing [21]. For instance, in many plant species, miRNA targeted genes are involved in a mechanistic network of plant P signaling, e.g. the miR399-PHR1-PHO2 regulon [22,23], the miR156-SPL3-Pht1;5 pathway [24] and the miR827-NLA-P_i_ homeostasis network [25]. On the other hand, the abundance of small transcribed RNAs with perfect complementarity to targets, also called small interfering RNAs (siRNAs), correlate with DNA methylation levels and crucially determine sequence specificity of chromosomal DNA methylation [26]. This phenomenon is called RNA-directed DNA methylation (RdDM) and describes the maintenance of “*de novo*” methylation of DNA with certain sequence identities by silenced RNA [27][10].

Irrespective of their heritability, epigenetic mechanisms, such as DNA methylation and gene silencing by miRNAs, seem to have crucial functions in regulating gene expression in response to biotic stress [28]. In addition, DNA methylation potentially regulates the expression of miRNAs under abiotic stress, such as temperature stress [29,30]. Upon loss of sequence-specific DNA methylation in mutants of DNA methyltransferases in *Arabidopsis*, massive decrease in small RNAs was encountered [31], while methylation and miRNAs expression in bisexual flower development correlated negatively in poplar [32].

Nevertheless, most research has focused on annual herbaceous plants, leaving perennials and vegetatively propagated plants, such as black cottonwood (*Populus trichocarpa*), less investigated. In perennials, DNA methylation and miRNAs may likely have different or additional roles than in annual plants. For example, the oil content of vegetatively propagated clonal oil palms is regulated by the abundance of DNA methylation: A certain hypomethylation pattern predicts parthenocarpy and therefore a dramatic loss of yield [33]. Furthermore, the analysis of clonal white poplar populations indicates a quite limited genetic biodiversity, but detects a highly variable epigenetic status, where environmental conditions are strongly linked to DNA methylation [34]. Additionally, the methylome of poplar has responded to drought stress [6,35]. Thus, seasonal and eco-site adaptation may potentially allow (reversible) adaptation to environmental constraints and seasonally “memorize” environmental conditions.

Stimulated by recent findings of transient massive DNA methylation in transposon sequences close to P_i_ starvation-induced genes in rice [5], we concentrated on DNA methylation, its impact on miRNAs expression and defined differential DNA methylation as an “epigenetic” mechanism, irrespective of its proof of inheritance. Hence, we asked the following research questions: Are there any epigenetic modifications due to previous different environmental conditions, like different P availabilities, that correlate with gene expression adaptations in perennial and clonal propagated poplar trees? Thus, it was analyzed whether clonal starting material (cuttings) from *Populus trichocarpa* (cv. Muhle Larson), which was harvested on two different locations with distinct P availability in northern Germany, showed “memory” with respect to their host site via differential, genome-wide DNA methylation. Whether methylation in coding regions, including differentially methylated miRNAs, lead to corresponding gene expression changes, was analyzed. Because of well described correlations between P availability and epigenetic modifications [5,20], a major focus was on the P nutrition background of these trees. By understanding site-specific and species-specific adaptations not only in their genetic, but also in their epigenetic aspects, the knowledge of epigenetic mechanisms and molecular interactions might be expanded. This may be useful in plant breeding or biodiversity studies in perennial vegetatively propagated plants.

## Materials and Methods

### Growth conditions

In spring 2013, 2014 and 2015, cuttings of *Populus trichocarpa* (cv. Muhle Larson) were harvested from two different short rotation forestry sites in Germany (Anderlingen [Ø 8.4°C, 750 mm] and Wallstawe [Ø 8.8°C, 582 mm]) [36]. The cuttings, which were 20 cm long and had a diameter of approximately 1 cm, were stored in a cold room (4°C) for a few weeks before planting. After each seasonal harvest and short storage, a surface disinfection was performed using 70% ethanol [37] and exposed cutting ends were sealed with paraffin wax. Subsequently, 10 cuttings per site and harvest period (in total: 60 cuttings during three years) were placed with bottom ends in tap water for 2 weeks to stimulate root development. Rooted cuttings were planted in 2.8-l pots filled with ¼ Hoagland solution in controlled climate chambers [38]: Air temperature was maintained at 22: 18°C (light, 16h: dark, 8h) with a photosynthetic photon flux density (PPFD) of approximately 100–150 μmol m^-2^ s^-1^. Air humidity was kept constant at a level of 55%. To generate subsequently P deficiency, plantlets were divided into two groups, which were subjected to the following nutrient supplies: adequate phosphorus (+P) treatment (¼ Hoagland solution was changed once per week, whereas P_i_ was supplied as 0.1 M KH_2_PO_4_) and deficient phosphorus (−P) treatment (¼ Hoagland solution was changed every week, whereas P_i_ was supplied as 0.01 M KH_2_PO_4_ supplemented with KCl to ensure all plants receive the same K amount).

### Nutrient, soil and statistical analysis

In addition to the measurement of the acid extractable P concentration of material from at least three seasonally harvested or cutting-derived plants per chosen sites (Anderlingen and Wallstawe), soil samples taken from the upper layer (A horizon) were used to determine P concentration (calcium acetate lactate (CAL) extract) [39], pH (CaCl_2_ suspension) and soil texture (sieving and filtering) to perform soil classification [40]. In the growth experiment, leaves, stems and roots of the two sites (Anderlingen & Wallstawe) and treatments (+P & −P) were harvested after plantlets reached a height of 50 cm for P analysis. Morphological and physiological root comparisons were performed via WinRHIZO (Regent Instrument Inc., Canada). Furthermore, P concentrations, morphological and physiological parameters were determined by analysis of variance (one-way or two-way ANOVA) and their means were compared via Tukey’s HSD-test (p ≤ 0.05). All statistics were performed using the R project for statistical computing.

### Whole genome bisulfite sequencing

Two replicates (five plantlets per replicate) from each site were used to determine their methylome. DNA was extracted only from young leaves using Qiagen’s DNeasy Plant Mini Kit according to manufacturer’s protocol due to minor expected tissue-specific DNA methylation differences between leaves and roots [41]. To generate the methylation pattern of clonal *Populus trichocarpa* leaf material derived from two different short rotation forestry sites (Anderlingen and Wallstawe), the library preparation was performed using the EpiGnome’s Methyl-Seq Kit with Qiagen’s EpiTect Bisulfite Kit used for sodium bisulfite conversion. Ultra-high-throughput paired-end (100 bp) sequencing was applied by using Illumina’s HiSeq 2000 platform following the manufacturer’s instructions and library spiking of 20% PhiX as internal standard. Raw data were processed according to EpiGnome’s Methyl-Seq Bioinformatics User Guide.

### Bioinformatics and mapping of BS-Seq reads

Poor quality reads and residual adapter sequences were filtered by Trimmomatic [42] and analyzed using the FastQC tool (Babraham Bioinformatics). In order to align the whole genome BS-Seq reads to reference sequences, the *Populus trichocarpa* genome (v2.0) provided by the National Center for Biotechnology Information [43] was converted and subsequently implemented for aligning BS-Seq reads using the Bismark tool [44]. Methylation calls and further analyses were genome-widely tracked and visualized by SeqMonk software (Babraham bioinformatics). Bisulfite conversion efficiency was calculated from unmethylated chloroplast sequence as a negative control following the formula: p = 1 –(# methylated cytosines) / (# of cytosines) [6,45] and amounted 99.17% (Anderlingen) and 99.11% (Wallstawe). Methylation distribution along single chromosomes was displayed with Bioconductor’s DMRcaller. Methylation levels around annotated transcriptional starting sites were identified using the R project for statistical computing. In addition, BS-derived differentially methylated regions (DMRs) were determined by the BSmooth algorithm and minimum absolute t-statistics using Bioconductor’s package bsseq [46]. Methylation calls with a minimum coverage of three reads per sample were included in further analysis. A mean difference across the DMR of at least 0.1 and a quantile-based cutoff of 0.025 and 0.0975 were chosen. The top 200 DMRs in every context (CpG, CHG and CHH) were analyzed, compared and visualized in detail to select annotated genes affected by differentially methylated states. For DMRs in promoter sequences, 2000 bp upstream of the open reading frame were considered. Overrepresentation gene analysis was performed via PopGenIE [47].

### Mature miRNA analysis

DMRs were analyzed using miRBase [48] to find mapped differentially methylated miRNA genes of *Populus trichocarpa*. Therefore, miRNAs from leaves and roots (+P & −P) were isolated via analytikjena’s innuPREP Micro RNA Kit following the standard protocol. The expression of the identified differentially methylated miRNAs was quantified via qPCR by addition of a poly-A-tail and a universal adapter following the instructions of Agilent‘s miRNA 1st-Strand cDNA Synthesis Kit. Forward primers were designed using the miRNA sequences of miRBase [48], whereas the universal reverse primer, annealing to the 5‘ end added universal adapter, was provided by the kit. Short, non-coding RNA quantification was performed using the Agilent 2100 Bioanalyzer and Agilent’s Small RNA Analysis Kit according to the standard protocol.

### Transcription analysis

Gene selection for transcription analysis was performed by including only DMRs which occur in gene and predicted promoter sequences in every context due to high probability of changes in gene expression related to DNA methylation. Additionally, target genes of differentially methylated miRNAs were identified via psRNAtarget tool with a maximum expectation value to score the complementarity between small RNA and their target transcript of 2.0 [49,50]. Therefore, RNA was extracted using analytikjena’s innuPREP RNA Kit according to manufacturer’s instructions. Afterwards, 1 μg of total RNA was reverse transcribed to cDNA via QuantiTect Reverse Transcription Kit.

PCR reactions were performed in 3 technical and 3 biological replicates using KAPA SYBR FAST Universal 2x qPCR Master mix. Primers used in qPCR for gene candidates were created by Primer-BLAST [51] with an estimated primer melting temperature (Tm) of approximately 60°C, whereas primers for reference genes were chosen according to Xu et al, 2011 [52] (S1 Table). Real-time qPCR was carried out using a two-step protocol in the Bio-Rad CFX96 instrument. In addition, the 2-ΔΔCT method was used to calculate the relative transcript levels by the Bio-Rad software [53].

Gene expression studies were obtained in leaves and roots of both treatments (+P & −P) harvested after plantlets reached a height of 50 cm. Target genes and differentially methylated genes were annotated via PopGenIE [47].

## Results

### Poplar establishment, roots and P nutrition at two distinct sites

**Fig 1:**
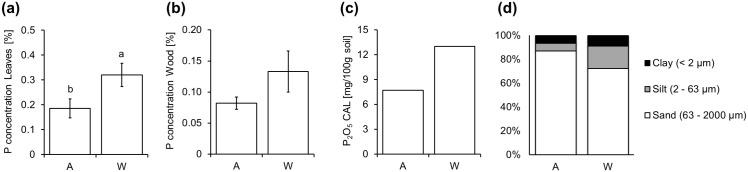
Phosphorus nutrition facts of two different sites. Acid extractable phosphorus (P) concentrations (y-axis) are given for leaf (a) and wood (b) material from clonal *Populus trichocarpa* (cv. Muhle Larson) harvested from two short rotation forestry sites (A = Anderlingen vs. W = Wallstawe; x-axis). P concentration (P_2_O_5_ in mg/100 g soil, extracted via calcium acetate lactate [CAL] method) in the soil of the two different sites (c) and their soil texture analysis by sieving and filtering (d) are additionally illustrated. P concentrations (a,b) were analyzed by one-way ANOVA and their means were compared via Tukey’s HSD-test (p ≤ 0.05). Data are presented as the mean ± standard error (SE) and 95% confidence intervals and were obtained from 3 independent measurements (a,b).

Cuttings and leaf samples of poplar clones (*Populus trichocarpa* cv. Muhle Larsen) were obtained from two short rotation forestry sites in Germany (Anderlingen and Wallstawe) [36]. Phosphorus concentrations in leaves, wood and soil, as well as the soil texture were measured and identified differences in the phosphorus background of the two chosen sites (Fig 1). Plant and soil material directly derived from Anderlingen always showed lower P concentrations (Fig 1a–1c). Therefore, Anderlingen was defined as a low P_i_ and Wallstawe as adequate P_i_ site, although Anderlingen material did not show severe P deficiency symptoms. According to VDLUFA, 2000 [40], soil texture analysis classified the soils from the two distinct sites as sandy (Anderlingen) and loamy sand (Wallstawe), indicating similar soil features in soil aeration, but an improved water and nutrient holding capacity in Wallstawe, due to a higher proportion of silt and clay (Fig 1d). The pH in 30 cm soil depth (B horizon) was determined as 5.9 in Anderlingen and 5.0 in Wallstawe. Both values were considered as acid soils, where P_i_ could be partially absorbed and fixed by aluminum.

**Fig 2:**
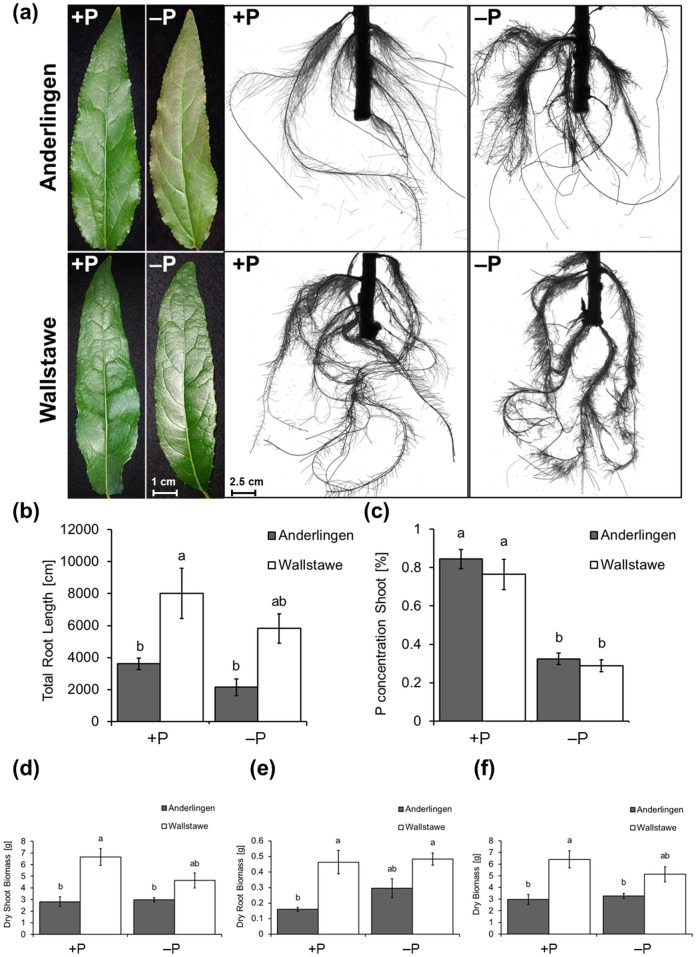
Site-dependent growth performance of *Populus trichocarpa* clones under different phosphorus nutrition levels. Leaf and root morphology (a), total root length in cm (b), percentage of P concentration in shoots (c), dry shoot (d), dry root (e) and total dry biomass (f) from clonal *Populus trichocarpa* (cv. Muhle Larson) derived from cuttings of two different short rotation forestry sites (Anderlingen vs. Wallstawe) under adequate (+P) and deficient (−P) phosphorus supply are illustrated. The different parameters (b-f) were analyzed by two-way ANOVA and their means were compared via Tukey’s HSD-test (p ≤ 0.05). Data are presented as the mean ± SEand 95% confidence intervals (b-f) and were obtained from 3 independent experiments.

Stem cuttings derived from these sites had slightly different P stored in the stem (Fig 1b), with cuttings from the low P_i_ site (Anderlingen) establishing worse than those from the high P_i_ site in a common environment in a plant growth chamber. For the analysis of establishment, only stem cuttings with identical diameter and length were chosen, to exclude size effects of the stems. This different establishment in nutrient solutions was observed both with adequate (+P) and low (−P) phosphorus supply (Fig 2). Interestingly, typical P deficiency symptoms (e.g. anthocyanin accumulation in leaves and short dense root system) were strongly visible in plants derived from cuttings from the lower P_i_ site, but not in plants established from cuttings from the high P_i_ site Wallstawe (Fig 2a), confirming their different P_i_ status. However, after establishment in the growth chamber, the P concentration in the shoot did not depend on the origin of the cuttings; it was only dependent on the P_i_ supply by the nutrient solution (Fig 2c). By contrast, root architectural traits and the overall growth performance, determined by the shoot and root biomass production, differed significantly and depended on their origin, suggesting a site-dependent, but not necessarily a P-dependent “memory” effect for plant establishment (Fig 2b, 2d–2f).

### Site-dependent whole-genome methylome and context-specific methylation differences

**Fig 3:**
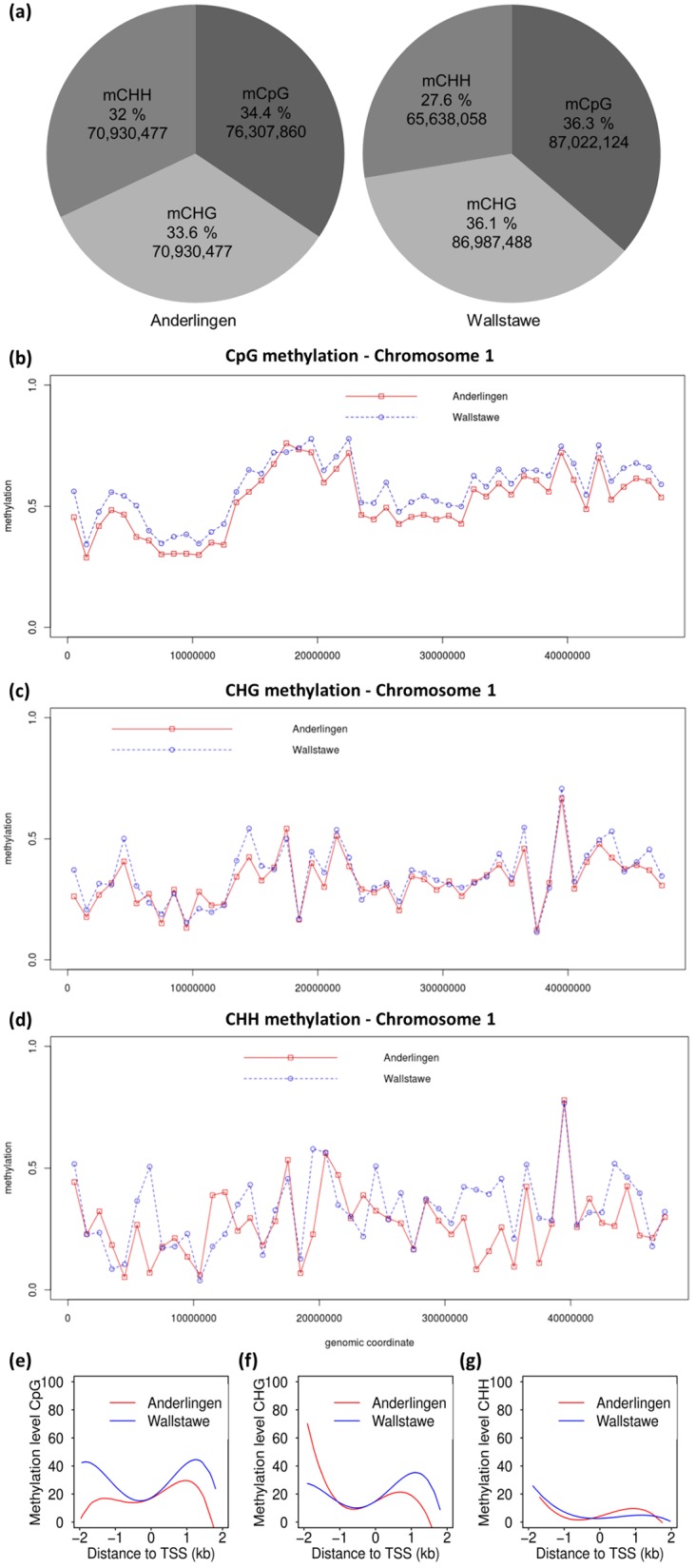
Methylation distribution in clonal *Populus trichocarpa* leaf material. Absolute methylation levels and absolute number of methylation (a) in the bisulfite sequenced data set, methylation distribution over chromosome 1 (b-d) and methylation distribution around transcriptional starting sites (TSS; e-g) of every cytosine context (CpG, CHG and CHH—where H represents the nucleotides A, T or C) in clonal *Populus trichocarpa* (cv. Muhle Larson) material derived from two different short rotation forestry sites (Anderlingen vs. Wallstawe) are illustrated. Distance to TSS is given in kilo base pairs (kb), whereas the promoter region is defined as -2 kb upstream and the gene body region as 2 kb downstream sequences.

Because of the identical, clonal genetic background of the cuttings and their differential performance despite identical, optimal, full nutrition, we considered that epigenetic differences might be causal for their differential performance. The whole genome DNA methylation pattern was derived via bisulfite sequencing only from leaf material from the low P_i_ (Anderlingen) and adequate P_i_ site (Wallstawe) due to minor expected tissue-specific DNA methylation differences between leaves and roots [41], after side-by-side establishment in the same growth chamber. DNA sequencing by the Illumina Hiseq 2000 platform yielded two sets of raw sequence data, with an output of 16.48 giga base pairs (Gb) in material derived from Anderlingen and 16.27 Gb in material derived from Wallstawe. The *Populus trichocarpa* genome (v2.0) served as a reference for mapping [43]. After applying several filter criteria (e.g. excluding poor quality reads or adapter sequence contaminations), it was possible to uniquely align 57% (Anderlingen) and 52% (Wallstawe) of the reads for further analysis. The estimated whole genome coverage of these data was 17.27 and 15.58, respectively (S2 Table). Besides, the bisulfite conversion efficiency rates were determined by the unmethylated chloroplast sequence and amounted 99.17% (Anderlingen) and 99.11% (Wallstawe), respectively. High quality data were obtained with minor global differences between the sites and identified a level of methylation of around 20% (S1 Fig). A slightly higher, but not significantly different amount of methylated cytosines (^m^Cs) in the whole genome was uncovered in plants derived from the high P_i_ site Wallstawe (20% vs. 17%). Furthermore, relative and absolute whole genome cytosine methylation differences in ^m^CpG, ^m^CHG and ^m^CHH context were encountered (Fig 3a and S2 Fig). The absolute methylation levels differed the most in the asymmetric CHH context (32% vs. 27.6%), suggesting a massive site-dependent difference in “*de novo*” methylation between the sites (Fig 3a).

When analyzed for individual chromosomes, the methylation distribution indicated high ^m^CpG densities in the assumed centromeric, non-coding heterochromatic region, which is shown as an example for the largest chromosome #1 (Fig 3b). ^m^CpG was gradually lower along the entire chromosome in Anderlingen material, while a similar ^m^CHG pattern was found along chromosome #1 (Fig 3c). CHH methylation, by contrast, was more variable, with regions of higher and lower ^m^CHH along the chromosomal axes (Fig 3d) of Anderlingen or Wallstawe material. These chromosomal maps indicated site-specific methylomes, with a general decrease in conserved ^m^CpG methylation in Anderlingen and more variable “*de novo*” methylation in the clones.

The methylation in each context was quantified around transcriptional starting sites (TSS). Relatively low methylation was revealed at the TSS and in neighboring upstream sequences, independent from the cytosine context, confirming previous results (Fig 3e–3g). Furthermore, higher CpG methylation in promoter regions (2 kb upstream; Fig 3e) and higher CHG methylation in the gene body regions (2 kb downstream; Fig 3f) were preferentially observed in plants derived from Wallstawe (Fig 3a–3c). However, the overall CHH methylation level around TSS did not differ in material derived from the two sites (Fig 3g). The overall higher methylation in seedlings from the P-adequate site Wallstawe was thus due to higher methylation in the CpG context (Fig 3a and 3b).

**Fig 4:**
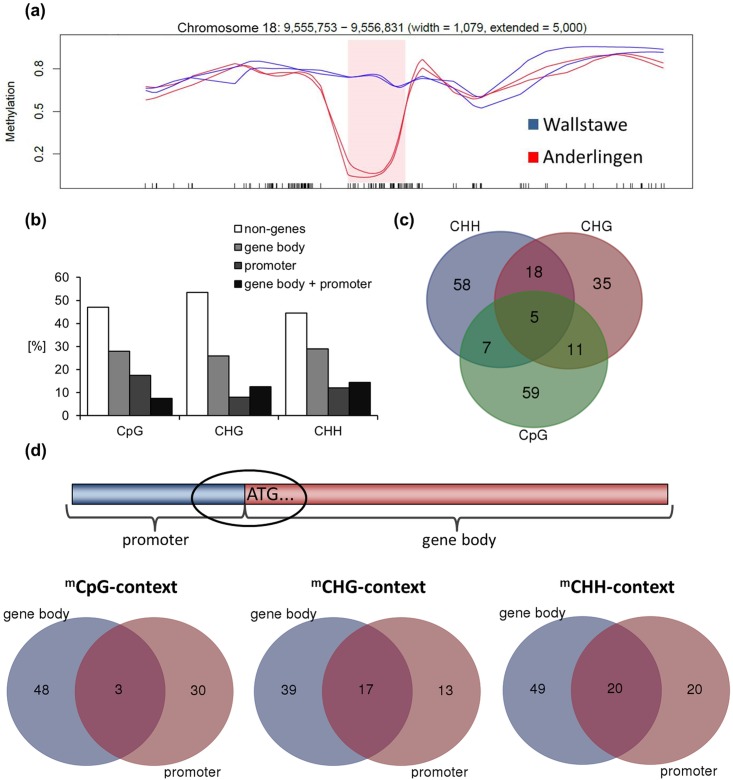
Differentially methylated regions (DMRs) and methylation context statistics. Pink shade (a) shows one of the top 200 DMRs in a CpG context between clonal *Populus trichocarpa* (cv. Muhle Larson) leaf material derived from two different short rotation forestry sites (Anderlingen vs. Wallstawe), x-axis represents CpG events (black bars) in the defined chromosome area (header) and the smoothed methylation level is shown on the y-axis (0 = no methylation; 1 = fully methylated sequence area). The base pair (bp) length of the illustrated DMR (width = 1,079 bp) is noted in the header, as well as the extended chromosomal area (5,000 bp) surrounding the DMR. (b) Proportion of DMRs (y-axis) in coding and non-coding regions for every methylated cytosine context (CpG, CHG and CHH—where H represents A, T or C; x-axis). (c) Venn-Diagram of all annotated differentially methylated genes shared in every cytosine context (CHH—violet, CHG—pink and CpG—green). (d) Schematic drawing of a promoter (blue) next to a gene body region (red), where ATG shows the start codon of a gene and the circuited area represents the selection of differentially methylated genes analyzed by qPCR, this selection is shown in the overlapping areas of the 3 Venn-diagrams in every methylated cytosine (^m^C) context (violet = DMRs in gene body, red = DMRs in promoter, pink = DMRs in promoter and gene body region).

After determination of the methylation status and distribution in the two data sets derived from the low P_i_ and adequate P_i_ site, thousands of differentially methylated regions (DMRs) in every context (CpG, CHG and CHH) were identified, using a mean difference across the DMR of at least 0.1 [46]. For further qPCR analyses, only the top 200 DMRs were selected. Two examples of chromosomal regions with differential methylation are given in Fig 4a and in S3 Fig. About half (~ 50%) of these DMRs were covering non-gene coding regions, potentially including many transposons or sequences transcribing miRNAs (Fig 4b). Differentially methylated regions in the CpG, CHG and CHH context partially overlapped and covered promoters, gene bodies (Fig 4c), as well as stretches of promoter and gene body (Fig 4d). Thereby, “*de novo*” methylations (CHG and CHH) were dominating in stretches covering both promoter and gene body regions (Fig 4d). Clear differences in the absolute methylation level in the CHH context were observed, but not around transcriptional starting sites (Fig 3a, 3d, 3g and S2 Fig).

Further enrichment analysis of all identified differentially methylated coding regions (S3 Table) with PopGenIE [47] to assess overrepresented differentially methylated gene categories did not reveal functional enrichment classes, even not for P homeostasis.

### Gene expression in shoots and roots of differentially methylated genes

**Fig 5:**
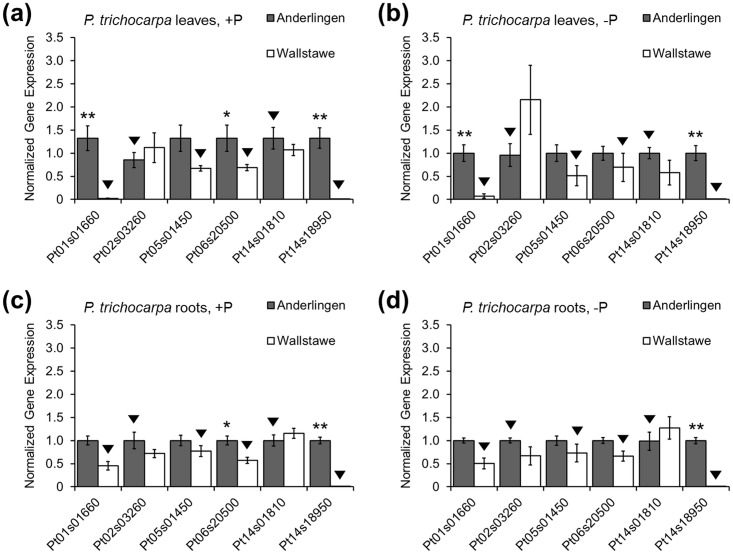
Gene expression differences of clonal *Populus trichocarpa* material. qPCR results from *Populus trichocarpa* (cv. Muhle Larson) leaf (a,b) and root (c,d) material derived from two different short rotation forestry sites (Anderlingen vs. Wallstawe), grown under controlled adequate (+P; (a,c)) and deficient (−P; (b,d)) phosphorus nutrition using 3 reference genes (*POPTR*_EF1α, *POPTR*_RP and *POPTR*_18s) for normalization. Normalized gene expression (y-axis) is shown for six differentially methylated genes (x-axis): *POPTR*_0001s01660g as Pt01s01660, *POPTR*_0002s03260g as Pt02s03260, *POPTR*_0005s01450 as Pt05s01450, *POPTR*_0006s20500g as Pt06s20500, *POPTR*_0014s01810g as Pt14s01810 and *POPTR*_0014s18950g as Pt14s18950. Black triangles indicate which plant material had a higher methylation level. Data are presented as the mean ± SEM, p* ≤ 0.05, p** ≤ 0.01 and 95% confidence intervals and were obtained from 3 independent experiments.

Since previous studies commonly identified a negative correlation of DNA methylation in promoter sequences and a strong negative correlation in promoter sequences immediately proceeding the genes in poplar [6], direct gene expression was then studied for the selected top 40 differentially methylated coding regions (S4 Table) in overlapping promoter and gene body regions (see overlapping regions in Fig 4d). However, only for 15% of these genes a reliable expression in either shoot or root was measured (Fig 5), suggesting that most of the top differentially methylated genes were either pseudogenes or not expressed during plant establishment. According to the annotation, none of these genes was functionally related to P acquisition or P metabolism, but because of the previously identified close correlation of DMR with phosphate-starvation-induced gene expression [5], we also checked gene expression in −P and +P conditions. The gene expression was not different between +P and the −P treatments (Fig 5a, 5c vs. 5b, 5d).

There was overall little difference between root and shoot gene expression of the selected top 40 genes (Fig 5a, 5b vs. 5c, 5d, only those six with reliable expression are shown). Of the six genes with substantial expression, three were repressed by methylation (*POPTR_*0001s01660, *POPTR_*0006s20500 and *POPTR_*0014s18950). A significant differential expression was only observed in a single case due to a differential symmetric CpG methylation. However, in the list of the top 40 DMR genes, “*de novo*” DNA methylations (CHH and CHG context) were much more prominent (Fig 4d and S4 Table). The six differentially methylated genes with reliable, but not always significantly different gene expression in leaves and roots were an NBS-LRR resistance gene-like gene (*POPTR_*0001s01660), a secretory peroxidase gene (*POPTR_*0002s03260), a PPR repeat family gene (*POPTR_*0005s01450), an elongation factor Tu family gene (*POPTR_*0006s20500), a peroxisomal membrane 22 kDa protein gene (*POPTR_*0014s01810) and a glycosyltransferase gene (*POPTR_*0014s18950).

Furthermore, the consequences of exclusive differences in DNA methylation just in promoter or just in gene body sequences were each quantified for three cases among the top 200 DMRs in leaves and roots via qPCR. Repression of gene expression by methylation in the promoter region was found only for one gene, while differential methylations exclusively in gene body regions did not significantly alter gene expression (S4 Fig and S5 Table). Overall, gene expression repression by methylation in promoter regions was identified, but rare, and found only in a minority of investigated gene sequences, questioning its importance for site of origin-dependent plant establishment in *Populus trichocarpa*.

### Methylation state in DMRs and transcript expression dynamics

**Fig 6:**
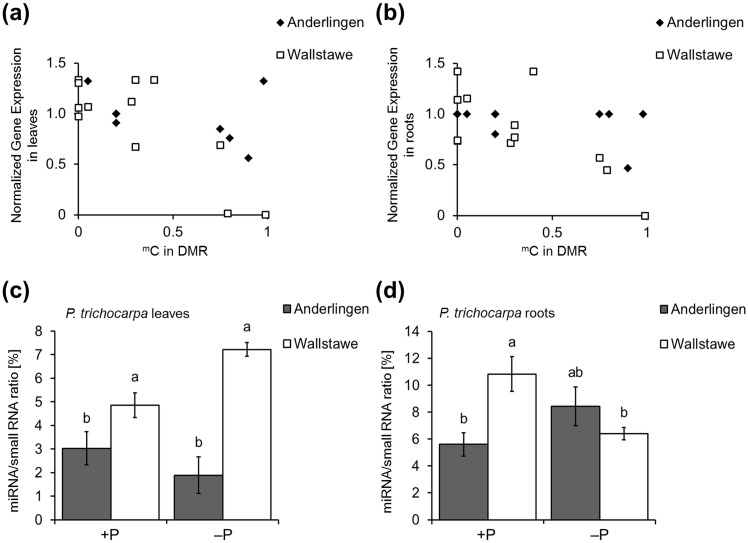
Gene expression vs. ^m^C state in DMRs and miRNA quantification of clonal *Populus trichocarpa* material. Shown are methylated cytosine (^m^C) states (0 = no methylation; 1 = fully methylated sequence area) in differentially methylated regions (DMRs) plotted against normalized gene expression (y-axis) of leaf (a) and root (c) material from clonal *Populus trichocarpa* (cv. Muhle Larson) cuttings derived from two different short rotation forestry sites (Anderlingen vs. Wallstawe), grown under optimal (+P) conditions. Additionally, quantification of miRNA/small RNA ratio in total RNA samples of leaf (c) and root (d) material from the same clonal *Populus trichocarpa* cuttings, grown under adequate (+P) and deficient (−P) phosphorus supply, are illustrated. Data are presented as the mean ± SE, p ≤ 0.05 and 95% confidence intervals (c,d) and were obtained from 3 independent experiments.

The normalized expression of the genes described above in leaves or roots was then plotted against their relative methylation level for material from both sites (Fig 6a and 6b). In leaves and roots, very high methylation repressed gene expression only in material from the adequate P site Wallstawe, but not in material from Anderlingen (Fig 6a and 6b). For Wallstawe cuttings, a negative and significant linear correlation between methylation levels in differentially methylated coding regions and gene expression was identified (Pearson’s product-moment correlation coefficient r = -0.80 for leaves (+P) and r = -0.71 for roots (+P), S6 Table). However, for Anderlingen cuttings, the ^m^C state was less important for gene expression (Pearson’s product-moment correlation coefficient was r = -0.57 (leaves) and r = -0.39 (roots), respectively), showing a less negative and not significant linear correlation between methylation amount and gene expression (S6 Table). Thus, for the small set of genes quantified here, the methylation level influenced gene expression stronger in plants derived from Wallstawe than in plants from Anderlingen, suggesting another level of gene expression regulation, besides differential DNA methylation.

### miRNA quantification and expression differences in DMRs

Since non-coding short RNAs regulate gene expression via multiple pathways, we isolated and quantified miRNAs to investigate whether these, besides DNA methylation, might be altered in plant material from different origin. The amount of short RNAs differed significantly between plants derived from Anderlingen or Wallstawe, despite that these cutting-derived plants were grown under identical conditions in the growth chamber. The abundance of miRNAs in leaves was significantly down in material with “poor” Anderlingen site history (Fig 6c), irrespective whether the plants were cultivated under +P or −P conditions. Different P nutritional conditions were explicitly considered in this analysis, because of the numerous miRNAs involved in P status signaling in plants. In the roots under +P conditions, the total miRNA abundance was again higher in Wallstawe than Anderlingen material, but in −P, total miRNA levels were similar at a level of Anderlingen +P (Fig 6d). The overall higher CpG methylation status in Wallstawe is likely responsible for higher miRNA abundance in material from this origin, in agreement with the fact that miRNAs are essentially lost upon loss of CpG methylation in *met1* mutants of *Arabidopsis* [31].

MiRNA differences between cutting-derived plant tissue material from Anderlingen and Wallstawe might also differ due to altered processing or synthesis. The cleavage of double stranded RNA or pre-mature RNA to produce siRNAs and miRNAs is performed by Dicer and Dicer-like3 enzymes, but their expression levels did not differ between plant material derived from Anderlingen or Wallstawe (S5 Fig), suggesting that processing or synthesis of miRNA between plants originating from the two distinct sites was not caused by differential processing enzyme expression. Furthermore, Dicer gene homologs were not listed in the differentially methylated regions and therefore shared a similar methylation pattern in the two sites (S6 Fig).

**Fig 7:**
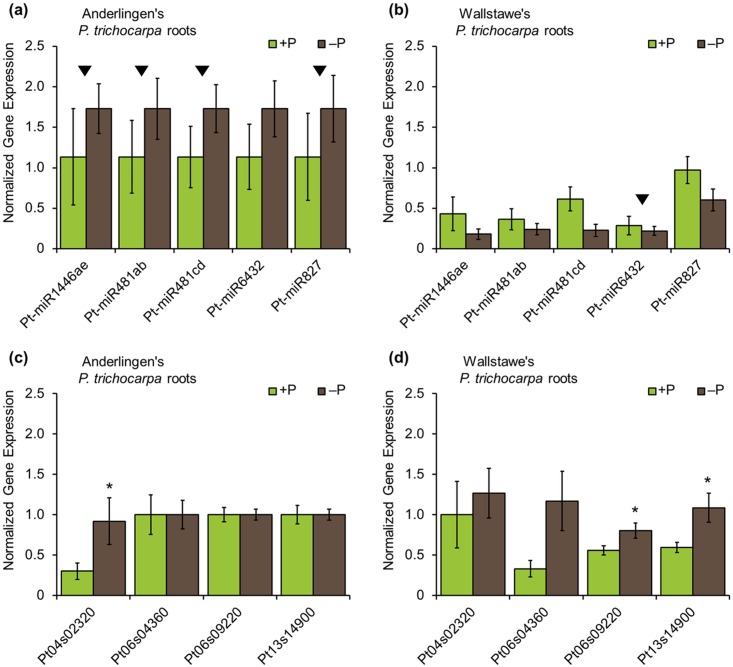
Target gene expression differences of clonal *Populus trichocarpa* material. qPCR results from Populus trichocarpa (cv. Muhle Larson) root material derived from two different short rotation forestry sites (Anderlingen (a,c) and Wallstawe (b,d)), grown under controlled adequate (+P) vs. deficient (−P) phosphorus nutrition using 3 reference genes (POPTR_EF103B0031, POPTR_RP and POPTR_18s) for normalization. Normalized gene expression (y-axis) is shown for differentially methylated miRNAs (a,b) and their possible target genes (c,d): Ptc-miR1446ae, Ptc-miR481ab, Ptc-miR4b1cd, Ptc-miR6432, Ptc-miR827, POPTR_0004s02320 as Pt04s02320, POPTR_0006s04360 as Pt04s04360, POPTR_0006s09220 as Pt06s09220 and POPTR_0013s14900 as Pt13s14900 (x-axis). Black triangles indicate which plant material had a higher methylation level. Data are presented as the mean ± SEM, p* ≤ 0.05 and 95% confidence intervals and were obtained from 3 independent experiments.

Finally, it was investigated whether differences in total miRNA expression were also reflected by differential expression of individual miRNAs in DMRs. Five miRNAs (*Ptc*-miR1446a-e, *Ptc*-miR481ab, *Ptc*-miR481cd, *Ptc*-miR6432 and *Ptc*-miR827) were encoded in the top 200 DMRs in every context (CpG, CHG and CHH). Their expression in material from both sites was quantified, to verify the relationship between DNA methylation and miRNA expression. Because of the crucial importance of several miRNAs for P-related signaling and the responsiveness of total miRNA amounts to P, +P and −P treatments were also investigated and are shown for the roots (Fig 7a and 7b; for leaf material see S8 Fig). The five miRNAs tested were highly expressed in root material derived from Anderlingen-derived cuttings, irrespective of the P supply. By contrast, these miRNAs were less expressed in roots derived from the P-adequate site Wallstawe, where their expression tended to be repressed in −P, in agreement with the total miRNA amounts (Fig 6). As expected, in four of the five cases, higher DNA sequence methylation coincided with higher miRNA abundance, but this was contrasted by *miRNA6432*, where the opposite was true. For the five miRNAs tested, their abundance in material from Wallstawe showed larger variance in their expression level with respect to P, similar to the expression described above for differentially methylated genes sequences (Figs 5, 6a and 6b). These miRNAs were apparently regulated in a P-nutrition-dependent way, especially in roots (Fig 7a, 7b and S7 Fig).

### Prediction of possible target genes and their expression

A large number of genes were potentially targeted by these differentially methylated miRNAs. The prediction strongly depended on the chosen maximum expectation value to score the complementarity between small RNA and their target transcript. The overrepresentation analysis of these target transcripts with a maximum expectation value of 3.0 with PopGenIE [47] did not detect any functional enrichment in metabolic pathways or functional gene classes. Because for two miRNAs no expression of the most stringent targeted genes was found, only the four most stringent possible target genes (maximum expectation value not higher than 2.0; *POPTR_*0004s02320g, *POPTR*_0006s04360g, *POPTR*_0006s09220g and *POPTR*_0013s14900g) with expression in roots were further analyzed (Fig 7 and S7 Table). These encode a serine/threonine kinase (*POPTR_*0004s02320g), an acetyltransferase family protein (*POPTR*_0006s04360g), a prenylated rab acceptor family protein (*POPTR*_0006s09220g) and an N-acetyltransferase (*POPTR*_0013s14900g). In Anderlingen roots, the serine/threonine kinase transcript, targeted by *Ptc*-miR6432, was differentially expressed between +P and −P, while the other genes were not affected by P. By contrast, the other three genes were repressed under +P in Wallstawe, in accordance with inverse abundance of the targeting miRNAs (Fig 7c and 7d). Similar effects were observed for gene expression in the leaves (S8 Fig), showing that miRNA and target expression correlated largely inversely in a P-related way (Fig 7 and S8 Fig). Although it cannot be excluded that other targets of the identified DMR-regulated miRNAs exist and were not identified, the differential methylation of miRNAs apparently primarily affected plants derived from the P-adequate and ^m^CpG-rich site Wallstawe, in an organ-specific and P-related way.

## Discussion

### Growth performance and P in material from two different sites

Generally, better nutrition improves plant growth and higher P supply increased the growth performance of different poplar varieties [54]. The maximum P_i_ uptake is typically found within a pH range of 5–6 [55], which matches the pH of the analyzed sites. Sufficient P supply and non-limiting growth is encountered with 0.18–0.30% P in mature poplar leaves [56]. The leaf P concentration from Anderlingen was at the lower end of these values, suggesting that Anderlingen is at the threshold to a low P_i_ site, while Wallstawe was well P supplied (Fig 1a). Since P starvation reversibly induced DNA methylation in transposable elements close to highly induced genes in plants, primarily in rice, but to a limited extent also in *Arabidopsis thaliana* [5], we considered the possibility that P-related differences might have occurred in plant material from the two distinct sites. Methylation changes in rice occurred after nearby gene transcription and could be partially propagated through mitosis, but no transgenerational inheritance was observed [5].

Besides P availability, water and temperature stress were previously correlated to DNA methylation changes in poplar and *Arabidopsis* [6,29,30]: In a global collection of *Arabidopsis thaliana* accessions, habitat temperature was negatively and precipitation positively correlated to DNA methylation [30]. The overall higher methylation level in Wallstawe opposes these trends from *Arabidopsis*, as annual precipitation was slightly higher and average temperature was slightly lower in Anderlingen (750 mm, 8.4°C), compared to Wallstawe (582 mm, 8.8°C). However, temperature stress changed the methylation pattern of miRNA genes and thus their expression in *Populus simonii* [29], indicating a species-specific epigenetic adaptation. Nevertheless, no enrichment of differential methylated genes in functional classes of biological processes was identified, suggesting no over-representation of any stress and potentially explaining the lack of correlation of DNA methylation changes and environmental conditions.

### Site-dependent methylome and context-specific methylation differences

Throughout the study, a site-specific growth performance of clonal *Populus trichocarpa* plant material derived from two distinct sites was observed, which was independent from P nutrition (Fig 2). Because of the clonal origin of the material, this was likely due to site-specific methylation patterns in Anderlingen and Wallstawe (Figs 3 & 4a). Site-dependent adaptation in plant establishment of clonal white poplar was recently attributed to DNA methylation, suggesting an inclusion not only of genetic but also of epigenetic aspects in plant biodiversity studies of vegetatively propagated plant species [34]. The results above suggest that for vegetatively propagated poplar clones “*de novo*” methylation (^m^CHG and ^m^CHH) is of outmost importance for gene expression (via repression of gene expression in promoters). A more variable absolute number and chromosomal distribution of asymmetric CHH methylations between the plant material derived from Anderlingen or Wallstawe was encountered (Fig 3), although the relative and absolute amount of methylation was higher in the CpG context than in the CHG or CHH context (Fig 3a and S2 Fig). Similar results in ^m^C context proportions were obtained in *Arabidopsis* [57], but not in poplar under different water stress treatments, where methylation was concentrated in non-CpG contexts [6].

The genome-wide analysis of DNA methylation in every cytosine context revealed enrichments in hypervariable chromosome regions and in transposable elements (TEs) leading to their silencing [31,45,58–61]. This was partially confirmed by the highest proportion of DMRs occurring in non-gene regions (Fig 4b) and by a higher centromeric chromosomal ^m^CpG distribution (Fig 3b). However, the annotation of the heterochromatin and TEs in poplar is still not sufficiently advanced to capture all TEs [62]. Therefore, the analysis of DNA methylations in TEs and their silencing effect were not a focus of this study.

### Differentially methylated gene expression and their expression dynamics

The function of DNA methylation within promoters and coding regions in plants is still largely unknown, although the methylation in some promoter regions impairs transcription factor binding and thus impairs transcription [63]. The function of DNA methylation within coding regions in plants might prevent aberrant expression from intragenic promoters [59,64] or increase the splicing accuracy [65,66]. These assumptions have largely been confirmed in *Populus trichocarpa* under water stress, where the most significant gene expression changes occurred 100 bp upstream of the TSS [6]. Whether gene expression was altered due to DNA methylation differences was therefore analyzed for genes with DMRs occurring simultaneously in gene body and promoter sequences (Fig 4d). Only few differentially methylated genes with reliable expression in leaves and roots were encountered, but all followed a pattern of repression by methylation in the promoter or minor direct effects, irrespective of P (Fig 5). Though mutational changes seemed to be limited in vegetatively propagated perennials [34], they could not be excluded as a possible reason for the few observed significant gene expression differences. Nevertheless, the so far described strong correlation between DNA methylation in coding regions and gene expression profiles in *Arabidopsis* [4], rice [5] and poplar [6] under different stress conditions was only partially confirmed, when considering individual cases of differential methylated genes and their expression (Figs 5 & 6). Most importantly, a site-specific, but not P-related”memory”effect might be explained by differential DNA methylation.

Furthermore, the importance of differential DNA methylation in actively regulating gene expression has been questioned more recently [67], which is in agreement with the results reported here. In addition, previous studies on poplar described a more repressive effect on transcription by gene body methylation than by promoter methylation, which is in contrast to our findings and also in contrast to *Arabidopsis*, supporting diverse, species-specific gene regulation patterns by methylation [68].

### Expression of differentially methylated miRNAs

Clearly, the detection of a negative correlation between gene expression and methylation state in coding regions of clonal poplar material from Wallstawe, but not from Anderlingen, was an important observation (Fig 6a and 6b). Thus, there must be different regulatory levels of gene expression and their dependence on DNA methylation, e.g. by post-transcriptional gene silencing mechanisms, via pre-microRNAs from transposon sites or mature miRNAs [69,70]. A close correlation of DMR and individual gene expression was not observed and questions a causal relationship, which is in agreement with recent studies [30,67]. This novel link to P-nutrition, of DMR-derived miRNAs that respond to P nutrition, is especially interesting, as plant P signaling involves a mechanistic network of protein coding genes targeted by microRNAs, such as the *miR399-PHR1-PHO2* regulon, which was identified in many plant species, including poplar [22]. PHO2 is a ubiquitin-conjugating E2 enzyme for post-translational protein degradation [71] and controls P starvation response genes, such as *IPS1*, *PHT1;8* and *PHT1;9*. The phloem-mobile shoot to root signal for low shoot P includes *miR399*, which targets the major *PHR1* transcription factor gene. Overexpression of *miR399* phenocopies the response to low P_i_ in roots [72]. In addition, *miR156-SPL3-PHT1;5* pathways also constitute a component of the P deficiency-induced regulatory mechanism in *Arabidopsis*. During P starvation, *miR156* is induced and therefore its target *SPL3* is repressed. This influences the anthocyanin accumulation as well as the expression of high affinity PHTs, increasing the P_i_ uptake [24]. However, *miR399* and *miR156* were not among the differentially methylated miRNAs, excluding that these regulons are directly targeted by DNA methylation.

Furthermore, the low P-induced *miR827* targets *NLA*, which seems to be involved in the repression of P_i_ uptake, demonstrating that *miR827* and its target *NLA* have a crucial role in regulating P_i_ homeostasis [25]. Remarkably, the significantly different expressed *ptc*-*miR827* was a direct target of DNA methylation, confirming a site-specific and P-dependent adaptation via differentially methylated miRNAs. Although, the *miR827* family is conserved between rice, *Populus trichocarpa* and *Arabidopsis* [73], the predicted targets in all three species occur to have different functions, indicating a species-specific function of *miR827* [25,73].

Nevertheless, most of the novel identified DMR-regulated miRNAs appear to have no direct analog in *Arabidopsis* and to be involved in stress responses [73–75]. Additionally, previous expression analyses also suggested that the methylation pattern of identified miRNAs probably influences their expression [29,32]. Thus, the normalized miRNA expression level stayed similar in poplar clones derived from Anderlingen, independent from treatment or analyzed tissue, but not from Wallstawe (Fig 7a, 7b and S7 Fig). This observation was consistent with the gene expression levels of differentially methylated genes (Figs 5, 6a and 6b), suggesting a site-specific adaptation in miRNA and gene expression and therefore in plant establishment [34].

### miRNA target genes and their expression

Although minor effects of DNA methylation depending on P nutrition were previously described, the quantification of miRNA differed significantly between +P and −P treatments in root material from the two distinct sites (Fig 6d), indicating a direct P-related “memory” effect. Additionally, these results were consistent with the expression of differentially methylated miRNAs and their target genes, showing P-related expression differences in roots derived from the two short rotation forestry sites, especially from Wallstawe. Thereby, post-transcriptional gene silencing by miRNA would explain the down- or up-regulated expression of the target genes under different P nutrition states correlated with the expression pattern of the associated miRNAs (Figs 6d, 7b and 7d). Furthermore, in rice P starvation induced changes in DNA methylation [5] and in the *Arabidopsis* phosphate starvation response, genes were regulated by chromatin remodeling, an epigenetic mechanism, suggesting a direct, but species-specific link between P nutrition and epigenetic adaptation [20]. In poplar, this P-related species- and site-specific epigenetic modification might be the RNA interference by differential methylated miRNAs.

## Conclusions

**Fig 8:**
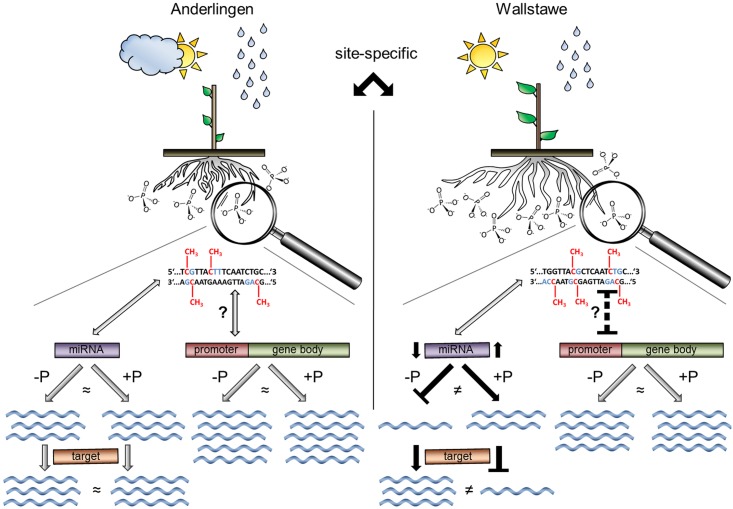
Schema of site-dependent differences in DNA methylation and their impact on plant establishment in clonal *Populus trichocarpa*. Two different short rotation forestry sites (Anderlingen vs. Wallstawe) are shown; where the same Populus trichocarpa clone (cv. Muhle Larson) is grown. Growth conditions differ on the two sites, e.g. phosphate availability. DNA nucleotides are presented as T (thymine), G (guanine), A (adenine) and C (cytosine). Analysis of the DNA methylation pattern (red in DNA sequence) has revealed differentially methylated regions (DMRs) in all C contexts (blue in DNA sequence) between plants derived from Anderlingen or Wallstawe. A causal relationship between DNA methylation in coding regions and gene expression changes still remains unclear: In Anderlingen plants, the methylation rate in coding regions has no effect on gene expression (blue waves), independent from optimal (+P) or deficient (−P) phosphorus nutrition. In Wallstawe plants, methylation level in coding regions is negatively correlated with the gene expression (indicated by black dashed T-shaped bar). This observation is independent from phosphorus nutrition (indicated by neutral grey arrows). Though, DNA methylation has a different impact on DMR-regulated miRNAs and their targets: In Anderlingen plants, miRNA expression is not related to phosphorus supply or DNA methylation. Therefore, gene expression of their targets in both conditions is not different. In Wallstawe plants, miRNA expression depends on the phosphorus nutrition. −P leads to lower DMR-regulated miRNA expression (indicated by black arrows) and thereby to higher target gene expression. +P leads to a higher DMR-regulated miRNA expression and thereby to a lower target gene expression (indicated by a black arrow and T-shaped bar).

In this research project, clonal *Populus trichocarpa* (cv. Muhle Larson) material derived from two different short rotation forestry sites in northern Germany with different environmental conditions, like P availability, was analyzed. The establishment of cuttings in full nutrition depended on their history, suggesting that epigenetic mechanisms might be involved. Indeed, genome-wide DNA methylation differences were identified. Expression analysis of differentially methylated genes and promoters showed a minor repression of DNA methylation in promoters and sites close to the transcriptional starting site on gene expression with overall little relation to the P supply. It is therefore possible that the massive transient DNA methylation close to genes related to P starvation, recently reported for nutrient-solution-grown rice [5], is not found in perennial poplar, but it is possible that in the native environments where the poplar plant material was derived from, other stresses were more pronounced and the low P stress was not sufficient to induce measurable changes in DNA methylation of P starvation-related genes. Nevertheless, differentially methylated miRNA sequences and their predicted targets indicated not only a site-dependent, but also P-related expression difference. Overall, the better nutrient-supplied plants derived from Wallstawe seemed to be more adaptive to environmental stresses, including P starvation, than Anderlingen (Fig 8). Thus, site-specific and species-specific epigenetic modifications might be responsible for different adaptations to low P. Such epigenetic aspects must be accounted for plant breeding and biodiversity studies, especially of vegetatively propagated perennials, like poplar.

## Supporting Information

S1 Fig Alignment and methylation statistics of whole genome bisulfite sequencing of clonal *Populus trichocarpa* leaf material. Alignment statistics and absolute percentage of methylated cytosines in the whole genome are given for bisulfite sequenced leaf material from clonal *Populus trichocarpa* (cv. Muhle Larson) cuttings derived from two different short rotation forestry sites (Anderlingen vs. Wallstawe).
(TIF)

S2 Fig Relative abundance of methylated cytosines in clonal *Populus trichocarpa*. The relative abundance (b) of methylated cytosines (^m^C) in every context (CpG, CHG and CHH—where H represents the nucleotides A, T or C) is identified in clonal *Populus trichocarpa* (cv. Muhle Larson) leaf material derived from two different short rotation forestry sites (Anderlingen vs. Wallstawe).
(TIF)

S3 Fig Differential methylated region (DMR) between two bisulfite sequencing data sets occurring in all cytosine contexts. Methylation calls of one of the top 200 DMRs in clonal *Populus trichocarpa* (cv. Muhle Larson) leaf material derived from two different short rotation forestry sites (Anderlingen vs. Wallstawe) are illustrated. DMR occurred in all cytosine contexts (CpG, CHG and CHH—where H represents the nucleotides A, T or C; y-axis) and around the gene *POPTR*_0017s04440. Grey spots indicate the methylation level. Besides, the sequence window (header) is stated in kilo base pairs (kbp) and the genomic coordinates are given in base pairs (x-axis).
(TIF)

S4 Fig Gene expression differences of differentially methylated promoter or gene body sequences in clonal *Populus trichocarpa* material. qPCR results from *Populus trichocarpa* (cv. Muhle Larson) leaf (a) and root (b) material derived from two different short rotation forestry sites (Anderlingen vs. Wallstawe), grown under optimal nutritional conditions, using 3 reference genes (*POPTR*_EF1α, *POPTR*_RP and *POPTR*_18s) for normalization. Normalized gene expression (y-axis) is shown for six differentially methylated genes (x-axis): *POPTR*_0008s20220 as Pt08s20220, *POPTR*_0010s11680 as Pt10s11680, *POPTR*_0012s04860 as Pt12s04860, *POPTR*_0017s02120 as Pt17s02120, *POPTR*_0017s14590 as Pt17s14590 and *POPTR*_0018s14780 as Pt18s14780. In the analyzed differentially methylated genes, Anderlingen plants always had a higher methylation level. Data are presented as the mean ± SEM, p* ≤ 0.05, p** ≤ 0.01 and 95% confidence intervals and were obtained from 3 independent experiments.
(TIF)

S5 Fig Endoribonuclease Dicer expression differences of clonal *Populus trichocarpa* material. qPCR results from *Populus trichocarpa* (cv. Muhle Larson) leaf (a,b) and root (c,d) material derived from two different short rotation forestry sites (Anderlingen vs. Wallstawe), grown under controlled adequate (+P; (a,c)) and deficient (−P; (b,d)) phosphorus nutrition using 3 reference genes (*POPTR*_EF1α, *POPTR*_RP and *POPTR*_18s) for normalization. Normalized gene expression (y-axis) is shown for endoribonuclease Dicer homologs (x-axis): *POPTR*_0018s30840 as DCL3 and *POPTR*_0002s182401 as Dicer. Data are presented as the mean ± SEM, 95% confidence intervals and were obtained from 3 independent experiments.
(TIF)

S6 Fig Methylation pattern of endoribonuclease Dicer homologs in clonal *Populus trichocarpa*. Methylation calls of all cytosine contexts (CpG, CHG and CHH—where H represents the nucleotides A, T or C; y-axis) are shown for Dicer gene homologs (a,b) in clonal *Populus trichocarpa* (cv. Muhle Larson) material derived from two different short rotation forestry sites (Anderlingen vs. Wallstawe). Grey spots indicate the methylation level. Besides, the sequence window (header) is stated in kilo base pairs (kbp) and the genomic coordinates are given in base pairs (x-axis).
(TIF)

S7 Fig miRNA expression differences of clonal *Populus trichocarpa* material. qPCR results from *Populus trichocarpa* (cv. Muhle Larson) leaf (a,b) and root (c,d) material derived from two different short rotation forestry sites (Anderlingen vs. Wallstawe), grown under controlled adequate (+P; (a,c)) and deficient (−P; (b,d)) phosphorus nutrition using 3 reference genes (*POPTR*_EF1α, *POPTR*_RP and *POPTR*_18s) for normalization. Normalized gene expression (y-axis) is shown for five differentially methylated miRNAs (x-axis): *Ptc*-miR1446ae, *Ptc-*miR481ab, *Ptc-*miR4b1cd, *Ptc-*miR6432 and *Ptc*-miR827. Black triangles indicate which plant material had a higher methylation level. Data are presented as the mean ± SEM, p* ≤ 0.05, p** ≤ 0.01 and 95% confidence intervals and were obtained from 3 independent experiments.
(TIF)

S8 Fig Target gene expression differences of clonal *Populus trichocarpa* leaves. qPCR results from *Populus trichocarpa* (cv. Muhle Larson) leaf material derived from two different short rotation forestry sites (Anderlingen vs. Wallstawe), grown under controlled adequate (+P; (a)) and deficient (−P; (b)) phosphorus nutrition using 3 reference genes (*POPTR*_EF1α, *POPTR*_RP and *POPTR*_18s) for normalization. Normalized gene expression (y-axis) is shown for four genes possibly targeted by differentially methylated miRNAs (x-axis): *POPTR*_0004s02320 as Pt04s02320, *POPTR*_0006s04360 as Pt04s04360, *POPTR*_0006s09220 as Pt06s09220 and *POPTR*_0013s14900 as Pt13s14900. Data are presented as the mean ± SEM, p* ≤ 0.05, p** ≤ 0.01 and 95% confidence intervals and are obtained from 3 independent experiments.
(TIF)

S1 Table Information about used primer sets. (PDF)

S2 Table Description of two bisulfite sequencing data sets of clonal *Populus trichocarpa* (cv. Muhle Larsen) derived from two short rotation forestry sites (Anderlingen vs. Wallstawe). (PDF)

S3 Table Description of differentially methylated regions (DMRs) occurring in annotated coding regions of the *Populus trichocarpa* genome. (PDF)

S4 Table Description of differentially methylated regions (DMRs) occurring in annotated promoter and gene body sequences of the *Populus trichocarpa* genome. (PDF)

S5 Table Loci of differential DNA methylation in coding regions of clonal *Populus trichocarpa* derived from two short rotation forestry sites (Anderlingen vs. Wallstawe). (PDF)

S6 Table Pearson’s product-moment correlation of methylation state in differentially methylated genes and gene expression in plant material from clonal *Populus trichocarpa* (cv. Muhle Larson) cuttings derived from two different short rotation forestry sites (Anderlingen vs. Wallstawe). (PDF)

S7 Table Description of possible genes targeted by differentially methylated miRNAs in clonal *Populus trichocarpa*. Here, the maximum expectation value scores the complementarity between small RNA and their target transcript. With a lower maximum expectation value (0–2.0), a more stringent cut-off threshold and thereby a lower false positive prediction is set.
(PDF)
