# Supplementary figures and images for: Site-Dependent Differences in DNA Methylation and Their Impact on Plant Establishment and Phosphorus Nutrition in Populus trichocarpa
Source: PLoS One. 2016 Dec 19;11(12):e0168623. doi: 10.1371/journal.pone.0168623 (PMC5167412; doi:10.1371/journal.pone.0168623)

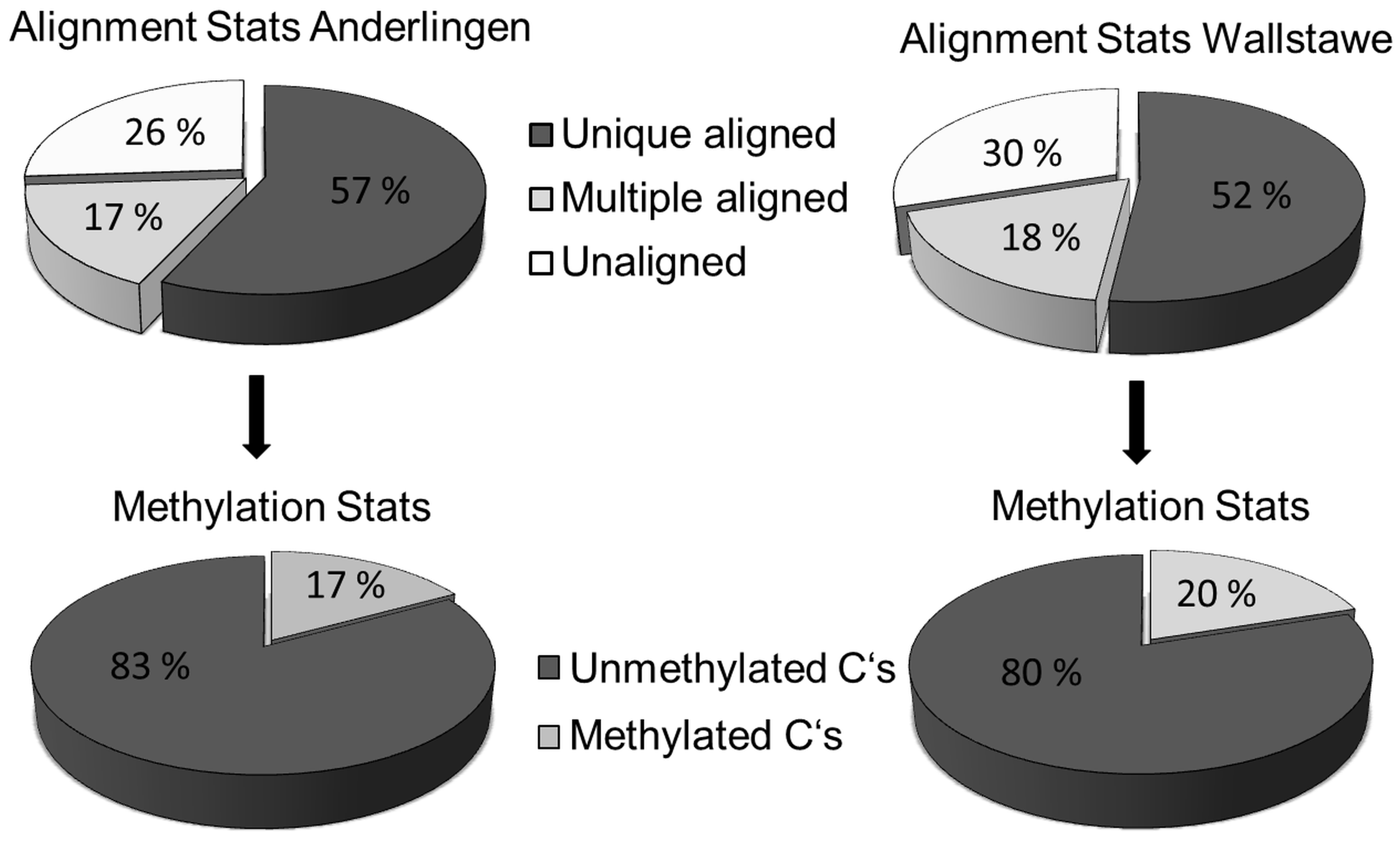

Supplement: S1 Fig — Alignment statistics and absolute percentage of methylated cytosines in the whole genome are given for bisulfite sequenced leaf material from clonal Populus trichocarpa (cv. Muhle Larson) cuttings derived from two different short rotation forestry sites (Anderlingen vs. Wallstawe). (TIF) [file pone.0168623.s001.tif]

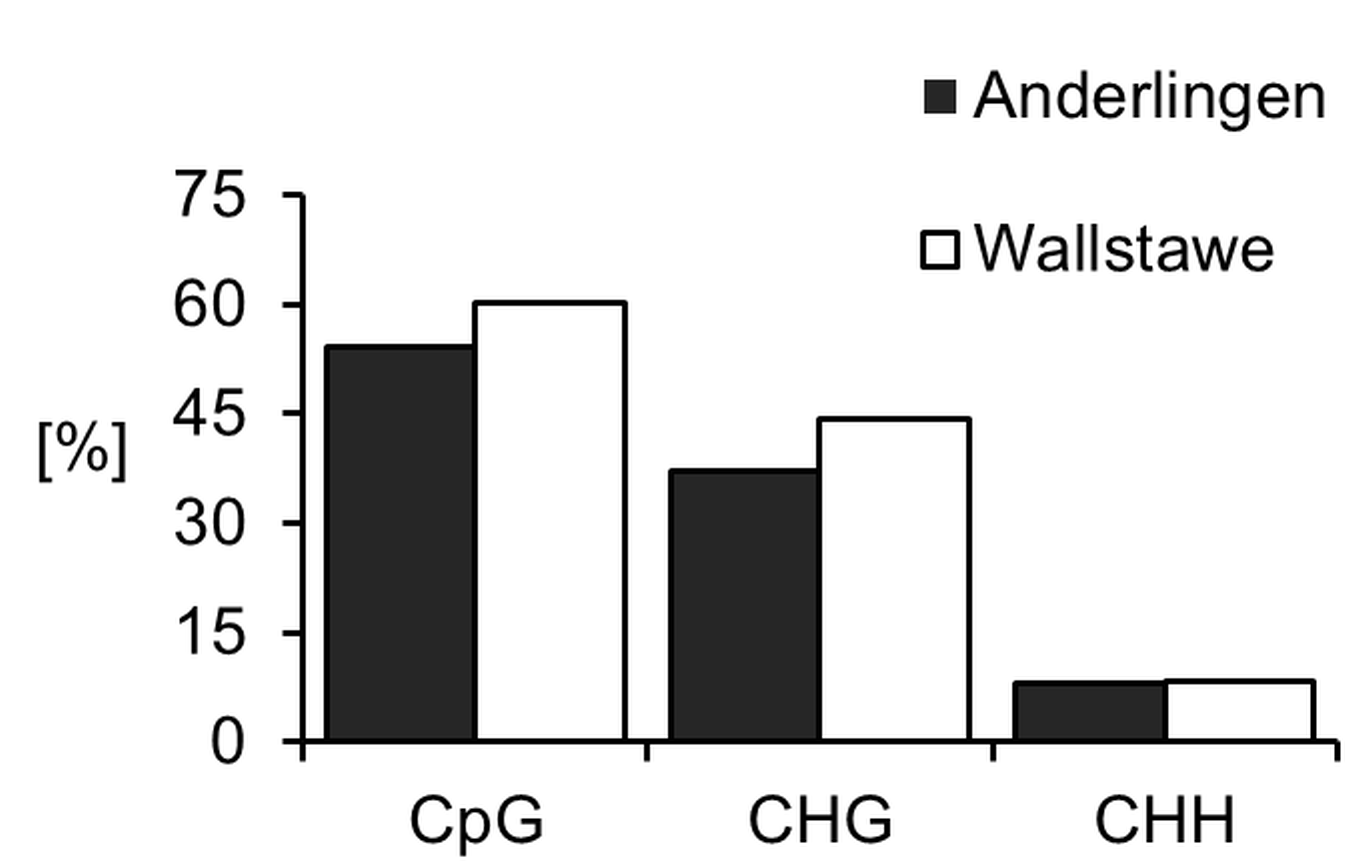

Supplement: S2 Fig — The relative abundance (b) of methylated cytosines (mC) in every context (CpG, CHG and CHH—where H represents the nucleotides A, T or C) is identified in clonal Populus trichocarpa (cv. Muhle Larson) leaf material derived from two different short rotation forestry sites (Anderlingen vs. Wallstawe). (TIF) [file pone.0168623.s002.tif]

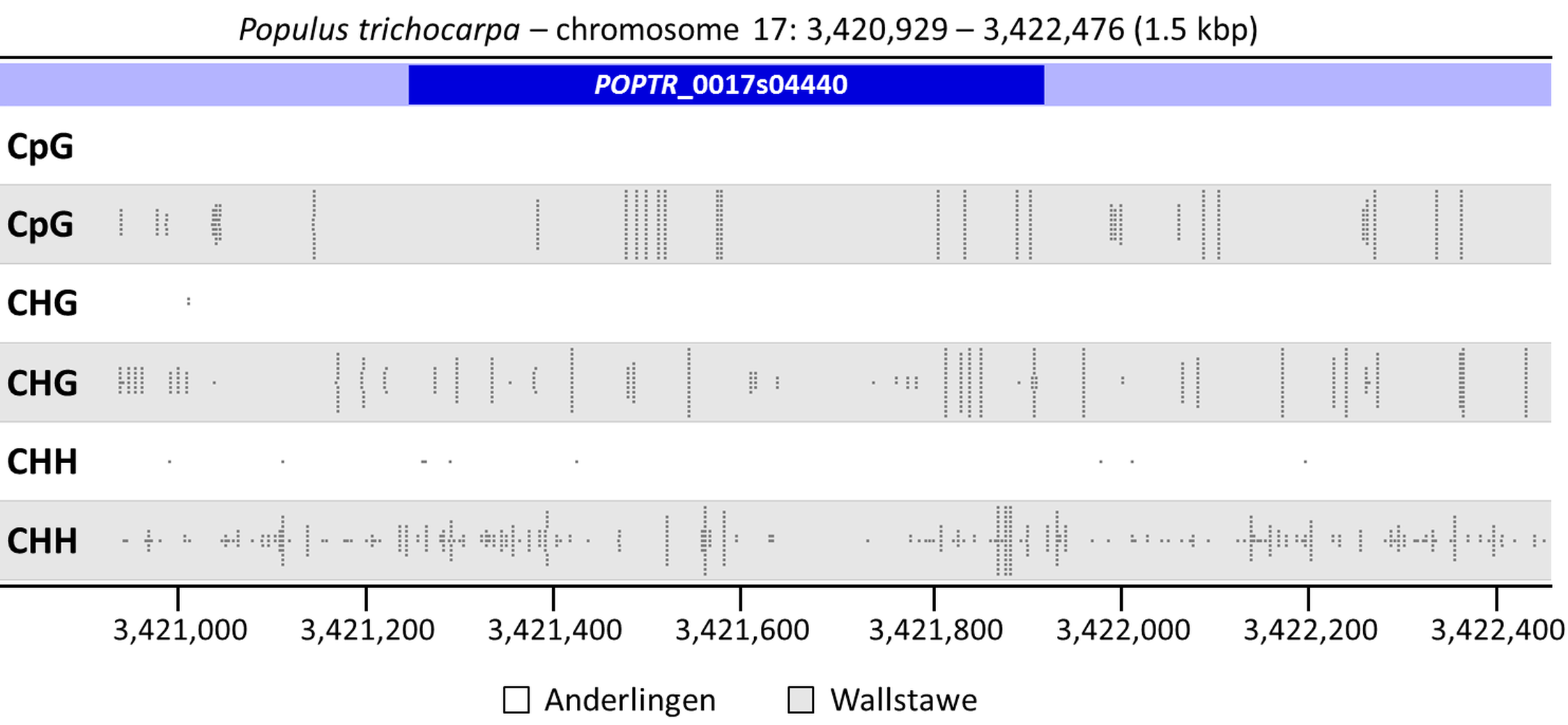

Supplement: S3 Fig — Methylation calls of one of the top 200 DMRs in clonal Populus trichocarpa (cv. Muhle Larson) leaf material derived from two different short rotation forestry sites (Anderlingen vs. Wallstawe) are illustrated. DMR occurred in all cytosine contexts (CpG, CHG and CHH—where H represents the nucleotides A, T or C; y-axis) and around the gene POPTR_0017s04440. Grey spots indicate the methylation level. Besides, the sequence window (header) is stated in kilo base pairs (kbp) and the genomic coordinates are given in base pairs (x-axis). (TIF) [file pone.0168623.s003.tif]

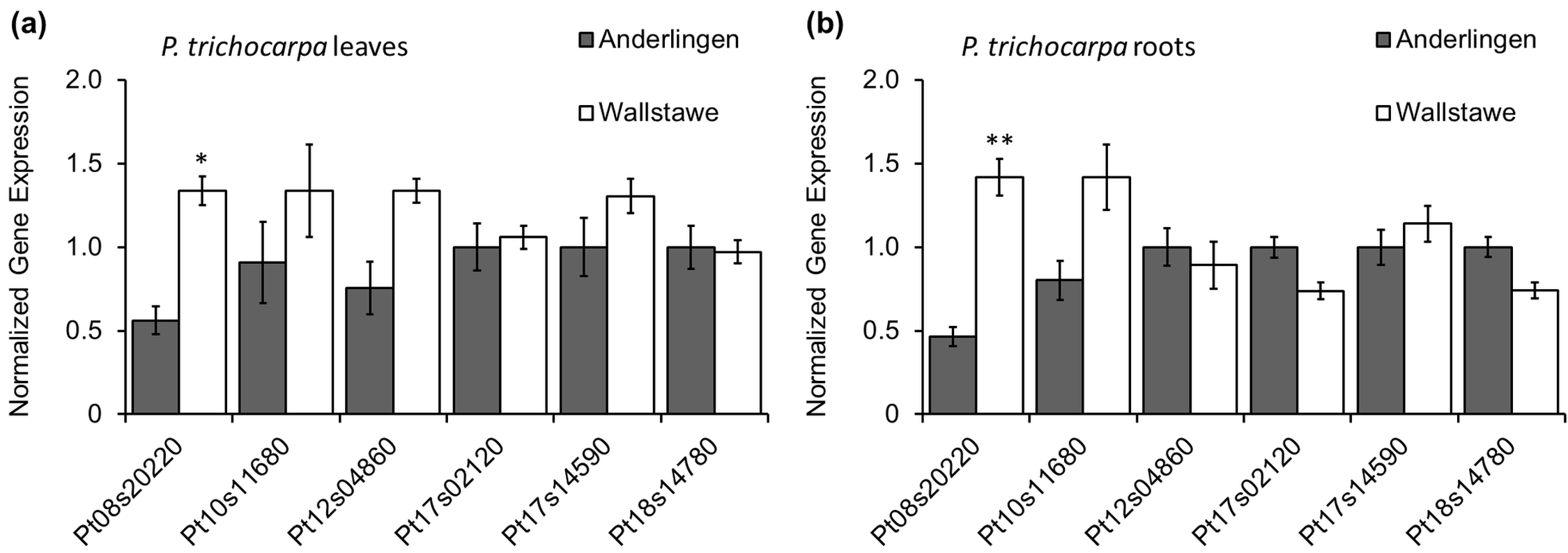

Supplement: S4 Fig — qPCR results from Populus trichocarpa (cv. Muhle Larson) leaf (a) and root (b) material derived from two different short rotation forestry sites (Anderlingen vs. Wallstawe), grown under optimal nutritional conditions, using 3 reference genes (POPTR_EF1α, POPTR_RP and POPTR_18s) for normalization. Normalized gene expression (y-axis) is shown for six differentially methylated genes (x-axis): POPTR_0008s20220 as Pt08s20220, POPTR_0010s11680 as Pt10s11680, POPTR_0012s04860 as Pt12s04860, POPTR_0017s02120 as Pt17s02120, POPTR_0017s14590 as Pt17s14590 and POPTR_0018s14780 as Pt18s14780. In the analyzed differentially methylated genes, Anderlingen plants always had a higher methylation level. Data are presented as the mean ± SEM, p* ≤ 0.05, p** ≤ 0.01 and 95% confidence intervals and were obtained from 3 independent experiments. (TIF) [file pone.0168623.s004.tif]

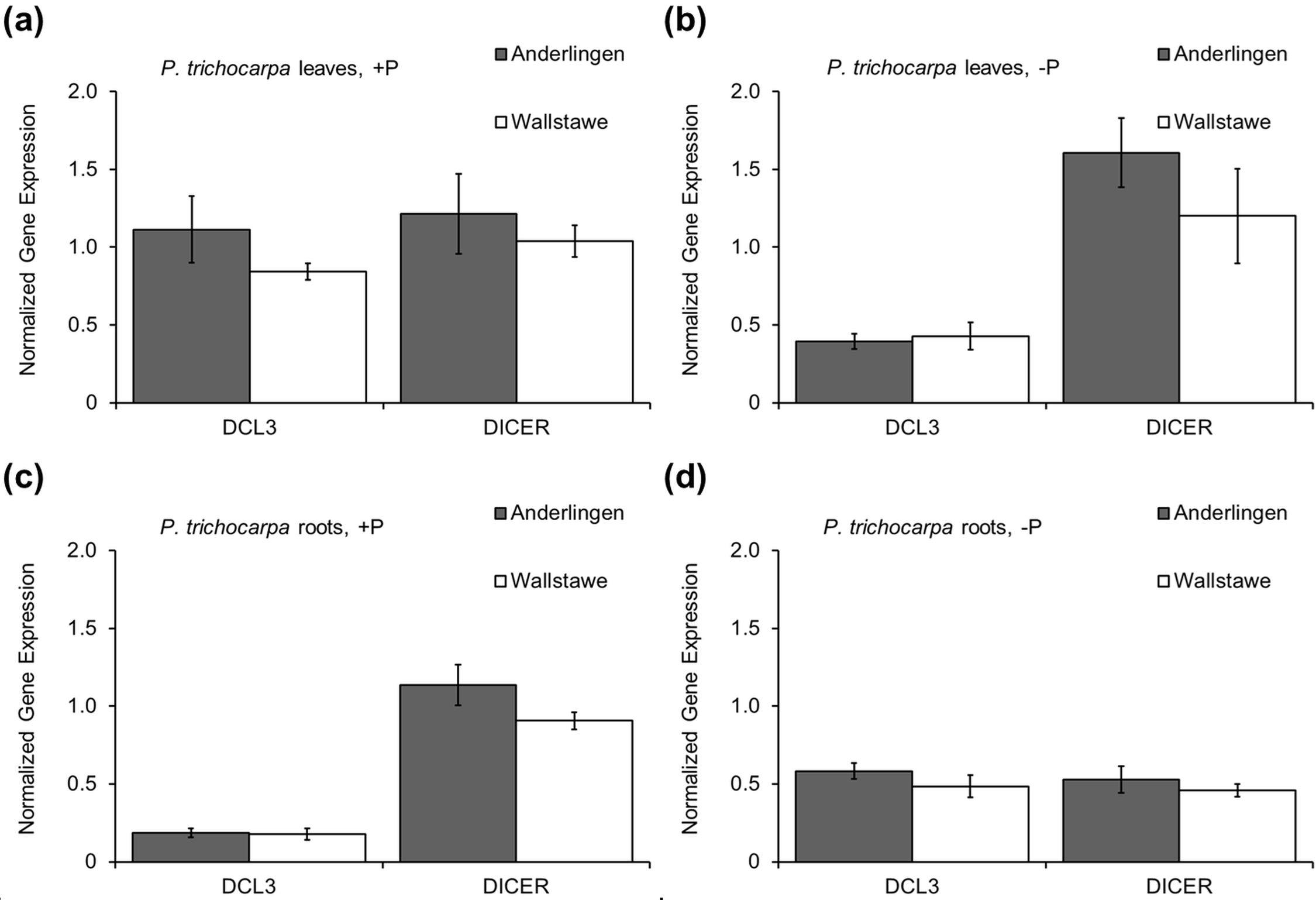

Supplement: S5 Fig — qPCR results from Populus trichocarpa (cv. Muhle Larson) leaf (a,b) and root (c,d) material derived from two different short rotation forestry sites (Anderlingen vs. Wallstawe), grown under controlled adequate (+P; (a,c)) and deficient (−P; (b,d)) phosphorus nutrition using 3 reference genes (POPTR_EF1α, POPTR_RP and POPTR_18s) for normalization. Normalized gene expression (y-axis) is shown for endoribonuclease Dicer homologs (x-axis): POPTR_0018s30840 as DCL3 and POPTR_0002s182401 as Dicer. Data are presented as the mean ± SEM, 95% confidence intervals and were obtained from 3 independent experiments. (TIF) [file pone.0168623.s005.tif]

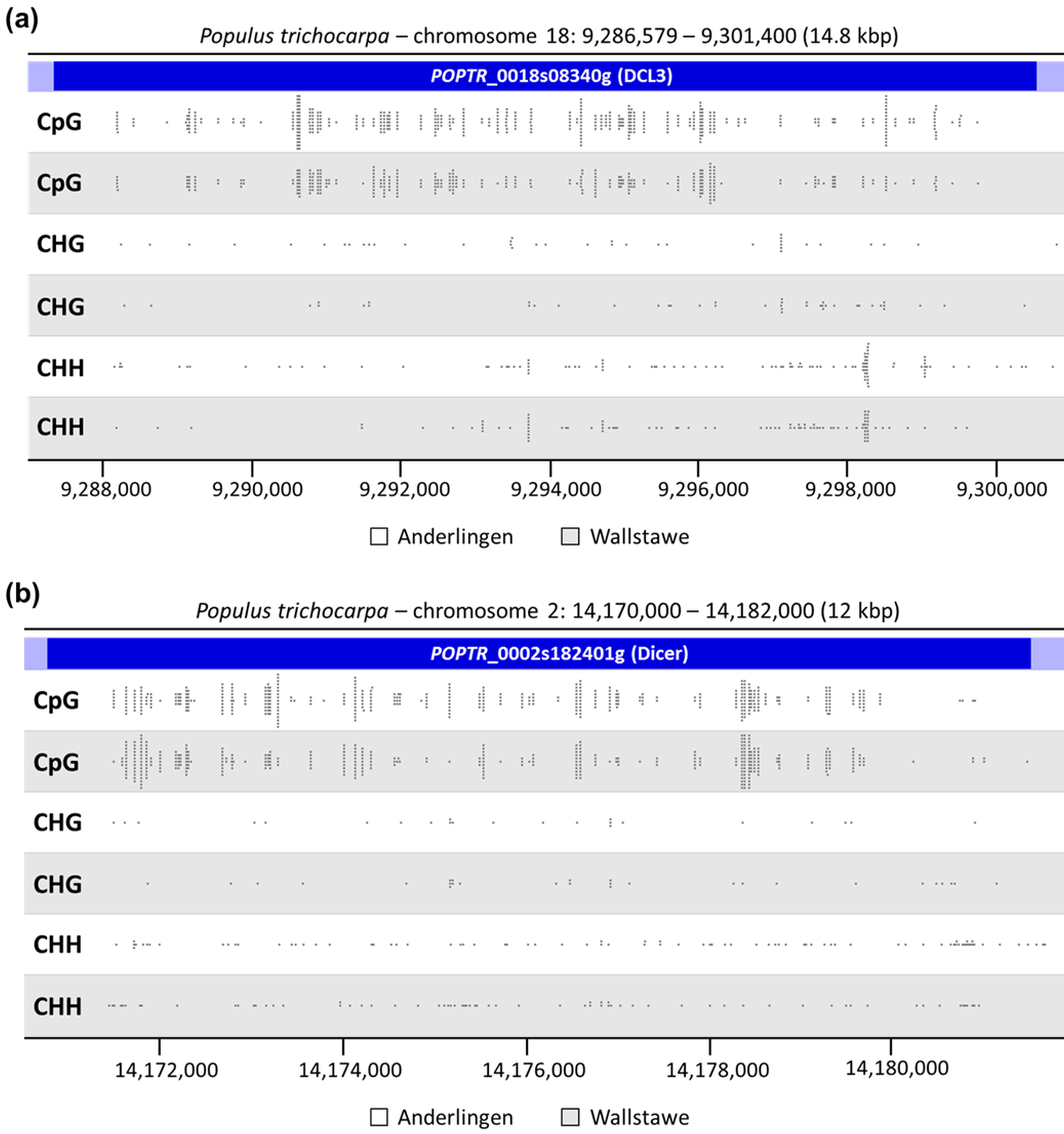

Supplement: S6 Fig — Methylation calls of all cytosine contexts (CpG, CHG and CHH—where H represents the nucleotides A, T or C; y-axis) are shown for Dicer gene homologs (a,b) in clonal Populus trichocarpa (cv. Muhle Larson) material derived from two different short rotation forestry sites (Anderlingen vs. Wallstawe). Grey spots indicate the methylation level. Besides, the sequence window (header) is stated in kilo base pairs (kbp) and the genomic coordinates are given in base pairs (x-axis). (TIF) [file pone.0168623.s006.tif]

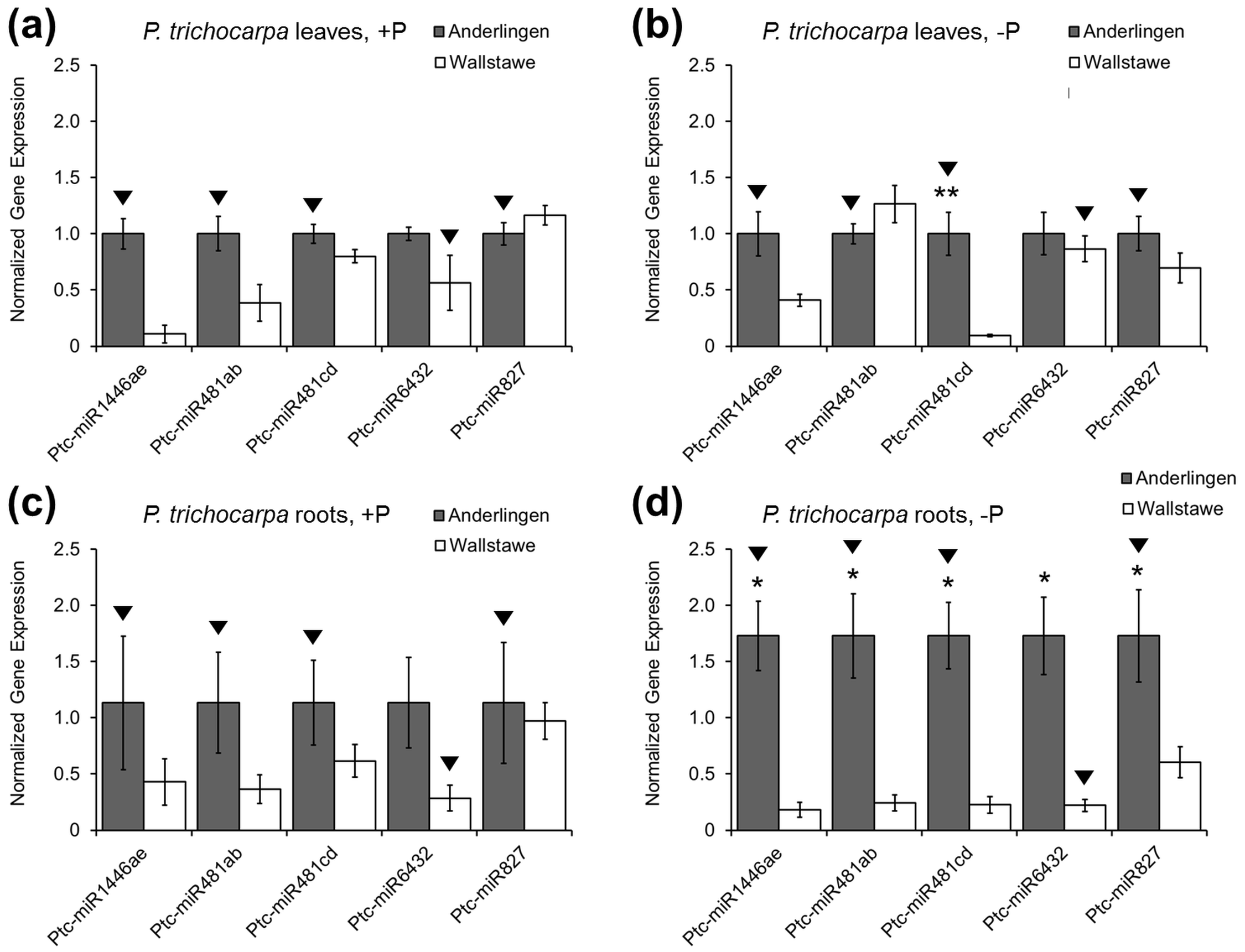

Supplement: S7 Fig — qPCR results from Populus trichocarpa (cv. Muhle Larson) leaf (a,b) and root (c,d) material derived from two different short rotation forestry sites (Anderlingen vs. Wallstawe), grown under controlled adequate (+P; (a,c)) and deficient (−P; (b,d)) phosphorus nutrition using 3 reference genes (POPTR_EF1α, POPTR_RP and POPTR_18s) for normalization. Normalized gene expression (y-axis) is shown for five differentially methylated miRNAs (x-axis): Ptc-miR1446ae, Ptc-miR481ab, Ptc-miR4b1cd, Ptc-miR6432 and Ptc-miR827. Black triangles indicate which plant material had a higher methylation level. Data are presented as the mean ± SEM, p* ≤ 0.05, p** ≤ 0.01 and 95% confidence intervals and were obtained from 3 independent experiments. (TIF) [file pone.0168623.s007.tif]

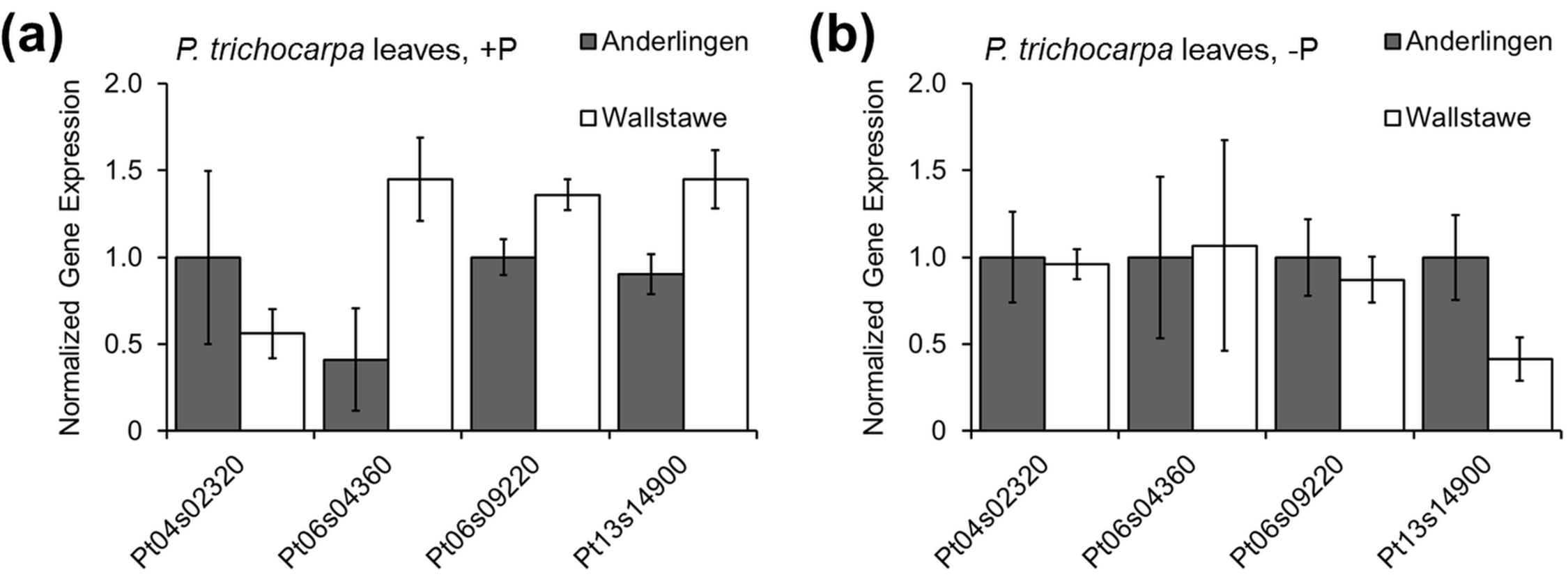

Supplement: S8 Fig — qPCR results from Populus trichocarpa (cv. Muhle Larson) leaf material derived from two different short rotation forestry sites (Anderlingen vs. Wallstawe), grown under controlled adequate (+P; (a)) and deficient (−P; (b)) phosphorus nutrition using 3 reference genes (POPTR_EF1α, POPTR_RP and POPTR_18s) for normalization. Normalized gene expression (y-axis) is shown for four genes possibly targeted by differentially methylated miRNAs (x-axis): POPTR_0004s02320 as Pt04s02320, POPTR_0006s04360 as Pt04s04360, POPTR_0006s09220 as Pt06s09220 and POPTR_0013s14900 as Pt13s14900. Data are presented as the mean ± SEM, p* ≤ 0.05, p** ≤ 0.01 and 95% confidence intervals and are obtained from 3 independent experiments. (TIF) [file pone.0168623.s008.tif]
