# Supplementary material for: Site-Dependent Differences in DNA Methylation and Their Impact on Plant Establishment and Phosphorus Nutrition in Populus trichocarpa
Source: PLoS One. 2016 Dec 19;11(12):e0168623. doi: 10.1371/journal.pone.0168623 (PMC5167412; doi:10.1371/journal.pone.0168623)
Supplement: S1 Table — (PDF) [file pone.0168623.s009.pdf]

S1 Table. Information about used primer sets.

primer set	forward primer (5’ -> 3’)	reverse primer (5’ -> 3’)
**DMR[1] transcript**		
*POPTR*_0001s01660	CTGATGACCGGATCCCTACC	GCCTTGATGTGCCTCTACGA
*POPTR*_0002s03260	CTGGTCTGTTCCCTGGATTCT	CACAACCCTGCACAAAGCAA
*POPTR*_0005s01450	TAGTGGATGAGGGACACCGT	GCATACCTGCACGTCCTAGA
*POPTR*_0006s20500	TCCCTGGAGATCCCCTTGAT	TCTTCACTCATTCCCTTGCGA
*POPTR*_0008s20220	TCTAATCAGGTTGGAGCAACAGA	TCTAATCAGGTTGGAGCAACAGA
*POPTR*_0010s11680	TGAGAAGGCTGCACTGGATG	AGCCTCCTCACCTCTGTCAT
*POPTR*_0012s04860	GGAAGGGGCAGATCACGAAT	GCGACTAAACACGGATAGCCT
*POPTR*_0014s01810	GCGTGACCTCCTTCCAACTC	TGTTCACCAATGGCTGTGGAT
*POPTR*_0014s18950	CGGCGGAATATGTTGGTAGG	TGTTGGTAAACCTCCCTCTCA
*POPTR*_0017s02120	GGGATAATAACAGCGCTCTGGA	TCCCTGTGGAAGGCATTTCT
*POPTR*_0017s14590	TGGTTCCTGCAATGGCCTAC	ATCAAGTACTCTAGCAGCTATTCCC
*POPTR*_0018s14780	GCTCTTTCTCACTCGGTCCTT	AGCGTTTGGCCATTCTCCTT
**endoribonuclease *Dicer***		
*POPTR*_0018s30840	TCGCTCTGGGAAGGTCAATC	AACTGCGGACTGTCTTAGGC
*POPTR*_0002s182401	GGATAGACCGTACGAAGCAC	AGAATCGCACAACCAACCAG
**miRNA target**		
*POPTR*_0006s04360	ACGCAAGGGTTTTGGTCACT	TCCCTTATCACCCCAGCAGTA
*POPTR*_0006s9220	CATTGGACTTCTCGCCACCA	AATAATCCAGGCCACAAGCTAC
*POPTR*_0013s14900	AACTTGAAGGCTGTGCCGTA	ACGCCAGAATAGCTGAACCA
*POPTR*_0004s02320	TGTTGTTCTTCGAAGCAAGGT	CCAATTACTCCTGCCTTTAGGA

**primer set**	**forward primer (5' -> 3')**	**reverse primer (5' -> 3')**
**DMR[1] miRNA**		
Ptc-miR827	TTTTCGTTGATGGTCATCTAA	universal reverse primer (Agilent's miRNA 1st-Strand cDNA Synthesis Kit)
Ptc-miR1446a-e	TTCTGAACTCTCTCCCTCAA	
Ptc-miR481ab	GCTTAAGCTGTTAAGTGAGGTCCT	
Ptc-miR481cd	AGGACCTCACCTAACAGCTTAAGC	
Ptc-miR6432	CCATCTTTTTCTCTAGAGCCG	
**reference**		
*POPTR*_EF1α	GGCAAGGAGAAGGTTCACAT	CAATCACACGCTTGTCAATA
*POPTR*_RP	ATGTTGTGACCGCTGATTGTT	AAACCGCTCCTCGAACCTA
*POPTR*_18S	TCAACTTTCGATGGTAGGATAGAG	CCGTGTCAGGATTGGGTAATTT

[1] DMR = differentially methylated region
