# Supplementary material for: Site-Dependent Differences in DNA Methylation and Their Impact on Plant Establishment and Phosphorus Nutrition in Populus trichocarpa
Source: PLoS One. 2016 Dec 19;11(12):e0168623. doi: 10.1371/journal.pone.0168623 (PMC5167412; doi:10.1371/journal.pone.0168623)
Supplement: S2 Table — (PDF) [file pone.0168623.s010.pdf]

S2 Table. Description of two bisulfite sequencing data sets of clonal *Populus trichocarpa* (cv. Muhle Larsen) derived from two short rotation forestry sites (Anderlingen vs. Wallstawe).

sample	Anderlingen	Wallstawe
raw reads (Gb)	16.48	16.27
clean reads (Gb)	14.71	14.55
unique aligned reads (%)	57	52
unique aligned reads (Gb)	8.38	7.57
average coverage	17.27	15.58
conversion efficiency rate (%)	99.17	99.11
conversion error rate (%)	0.83	0.89
