# Supplementary material for: Site-Dependent Differences in DNA Methylation and Their Impact on Plant Establishment and Phosphorus Nutrition in Populus trichocarpa
Source: PLoS One. 2016 Dec 19;11(12):e0168623. doi: 10.1371/journal.pone.0168623 (PMC5167412; doi:10.1371/journal.pone.0168623)
Supplement: S3 Table — (PDF) [file pone.0168623.s011.pdf]

S3 Table. Description of differentially methylated regions (DMRs) occurring in annotated coding regions of the *Populus trichocarpa* genome.

DMR gene IDs	annotation	^{m}C loci	higher methylation site	^{m}C context
POPTR_0001s01660g	NBS-LRR resistance gene-like protein ARGH35	gene body + promoter	Wallstawe	CHH + CHG
POPTR_0001s017003g	leucine-rich repeat (LRR) protein	gene body	Wallstawe	CHH
POPTR_0001s03710g	NB-ARC domain	promoter (1584 bp)	Wallstawe	CHH
POPTR_0001s06110g	dirigent-like protein, regulates coupling of monolignol plant phenols to generate the cell wall polymers lignins and lignans that are involved in structural fortification and defense against pathogens	gene body + promoter; promoter	Wallstawe	CHH; CHG
POPTR_0001s11520g	potassium transporter 13	gene body	Wallstawe	CpG
POPTR_0001s11530g	potassium transporter	gene body	Wallstawe	CpG
POPTR_0001s11770g	ethylene-responsive transcription factor 2	promoter (1039 bp)	Wallstawe	CHH
POPTR_0001s14470g	F-box-like, phloem protein 2	promoter (1033 bp)	Anderlingen	CHH
POPTR_0001s15680g	crotonase/enoyl-coenzyme A (CoA) hydratase superfamily	promoter (803 bp)	Anderlingen	CHH
POPTR_0001s15740g	agenet-tudor-like domain present in plant sequences, chromatin assembly factor complex 1 subunit p60	promtor (1905 bp)	Anderlingen	CHH
POPTR_0001s15870g	pepsin-like aspartic proteases from plants	promoter (1693bp)	Anderlingen	CpG
POPTR_0001s16820g	PAUSED family protein	gene body	Anderlingen	CHG
POPTR_0001s17820g	DNA polymerase III	gene body + promoter	Wallstawe	CHG
POPTR_0001s19460g	zinc knuckle – zinc ion binding, nucleic acid binding	gene body + promoter	Anderlingen	CHG
POPTR_0001s21240g	chloroplast outer membrane translocon subunit family protein	promoter (362bp)	Anderlingen	CpG
POPTR_0001s21620g	Rix1 complex component involved in 60S ribosome maturation	promoter (333 bp)	Wallstawe	CHG
POPTR_0001s220902g	serine carboxypeptidase S28	gene body	Wallstawe	CpG
POPTR_0001s28070g	hydroxymethylglutaryl-CoA lyase	gene body	Wallstawe	CHH

POPTR_0001s28350g	DNA binding domains involved in the transcriptional regulation of key eukaryotic developmental processes	gene body	Anderlingen	CpG
POPTR_0001s29620g	target of rapamycin family protein	gene body	Wallstawe	CHG
POPTR_0001s31940g	glycosyltransferase family 28 C-terminal domain	promoter (862bp)	Wallstawe	CpG
POPTR_0001s33130g	lysophospholipid acyltransferases (LPLATs) of glycerophospholipid biosynthesis: MGAT-like	gene body	Wallstawe	CHH
POPTR_0001s37410g	reverse transcriptases (RTs) from retrotransposons and retroviruses	gene body	Wallstawe	CHG
POPTR_0001s37490g	ZIP zinc transporter	gene body	Anderlingen	CHH
POPTR_0001s42010g	catalytic domain of protein kinases	gene body + promoter; gene body	Wallstawe	CHH; CpG
POPTR_0001s44180g	formin homology 2 domain-containing family protein	gene body	Wallstawe	CHG
POPTR_0001s46630g	alpha-ketoglutarate decarboxylase	gene body	Anderlingen	CHG + CpG
POPTR_0002s03260g	horseradish peroxidase and related secretory plant peroxidases	gene body + promoter	Anderlingen	CHH + CHG
POPTR_0002s07850g	component of the thylakoid-localized Sec system involved in the translocation of cytoplasmic proteins into plastid	gene body + promoter	Wallstawe	CHG
POPTR_0002s13530g	PPPDE putative peptidase domain	gene body	Wallstawe	CpG
POPTR_0002s13560g	cytochrome P450 family protein	gene body	Anderlingen	CHH + CHG
POPTR_0002s15770g	porcine testicular carbonyl reductase (PTCR)-like	gene body	Wallstawe	CHH + CHG
POPTR_0002s15950g	C2 domain third repeat found in multiple C2 domain and transmembrane region proteins	gene body	Wallstawe	CpG
POPTR_0002s18400g	saccharomyces cerevisiae DCR2 phosphatase and related proteins, metallophosphatase domain	promoter (1645 bp)	Anderlingen	CHH

POPTR_0002s20620g	NADH-ubiquinone oxidoreductase-related family protein	gene body	Wallstawe	CHH
POPTR_0002s23200g	aconitate hydratase, encodes a aconitase that can catalyze the conversion of citrate to isocitrate through a cis-aconitate intermediate, indicating a role in the response to oxidative stress	gene body + promoter	Wallstawe	CHG
POPTR_0002s24770g	wound-response family protein	promoter (1674 bp)	Anderlingen	CpG
POPTR_0002s26350g	protein kinase superfamily protein	promoter (793bp)	Wallstawe	CpG
POPTR_0003s00520g	NLI interacting factor-like phosphatase	promoter (113 bp)	Anderlingen	CHG
POPTR_0003s02310g	alpha-crystallin domain (ACD) found in alpha-crystallin-type small heat shock proteins, and a similar domain found in p23 (a cochaperone for Hsp90) and in other p23-like proteins	promoter (1938bp)	Anderlingen	CpG
POPTR_0003s06000g	BCCP, acetyl-CoA carboxylase, biotin carboxyl carrier protein	gene body	Anderlingen	CHH
POPTR_0003s06000g	biotinyl-domain or biotin carboxyl carrier protein (BCCP) domain	gene body	Anderlingen	CHG
POPTR_0003s10790g	TITAN3 family protein, condensin complex components	gene body	Wallstawe	CpG
POPTR_0003s12600g	C2 domain-containing family protein	gene body	Wallstawe	CpG
POPTR_0003s12640g	glycosyl hydrolase family 32, beta-fructosidases	gene body + promoter; promoter	Wallstawe	CHH; CHG
POPTR_0003s12640g	GH32 B fructosidase, glycosyl hydrolase family 32, beta-fructosidases	promoter (787bp)	Wallstawe	CpG
POPTR_0003s12980g	ubiquitin-conjugating enzyme E2-21 kDa 3 family protein	promoter (1852 bp)	Anderlingen	CHG + CpG
POPTR_0003s16640g	putative zinc finger motif, C2HC5-type	gene body	Wallstawe	CHG
POPTR_0004s00680g	peptidases S8, S53 families	gene body	Anderlingen	CHH
POPTR_0004s02520g	cysteine-rich receptor-like protein kinase	gene body	Wallstawe	CHH

POPTR_0004s05180g	glutamate-gated kainate-type ion channel receptor	promoter (1209 bp); gene body	Wallstawe	CHG; CpG
POPTR_0004s08710g	tir-nbs-lrr resistance protein	gene body	Wallstawe	CHG
POPTR_0004s19540g	peptidase/protease-associated domain	gene body	Wallstawe	CHH + CHG
POPTR_0004s24010g	leucine-rich repeat receptor-like protein kinase	gene body	Wallstawe	CHH + CHG
POPTR_0005s01150g	leucine-rich repeat (LRR) protein	gene body	Anderlingen	CHH
POPTR_0005s01450g	PPR repeat family, DYW family of nucleic acid deaminases	gene body + promoter	Wallstawe	CHH
POPTR_0005s03560g	lipoxygenase	gene body	Wallstawe	CHH
POPTR_0005s06330g	mitogen-activated protein kinase	gene body	Wallstawe	CpG
POPTR_0005s08180g	leucine-rich repeat (LRR) receptor-like protein kinase	gene body	Anderlingen	CHH
POPTR_0005s08440g	NAC domain protein	gene body + promoter	Wallstawe	CHG
POPTR_0005s10180g	tonoplast monosaccharide transporter2	gene body	Anderlingen	CHH
POPTR_0005s10180g	major facilitator superfamily (MFS): secondary transporters that includes uniporters, symporters and antiporters	gene body	Anderlingen	CHG + CpG
POPTR_0005s11730g	cytochrome BC1 synthesis	gene body + promoter	Anderlingen	CHH
POPTR_0005s15370g	tyrosine kinase, catalytic domain	gene body	Wallstawe	CpG
POPTR_0005s18260g	protein kinase superfamily protein	promoter (1194bp)	Wallstawe	CpG
POPTR_0006s00560g	oligopeptide transporter protein	promoter (707 bp)	Wallstawe	CHH
POPTR_0006s00560g	OPT oligopeptide transporter protein	promoter (813bp)	Wallstawe	CpG
POPTR_0006s02510g	glutathione S-transferases	gene body + promoter	Anderlingen	CHH
POPTR_0006s02520g	probable cinnamyl alcohol dehydrogenase	gene body	Anderlingen	CHH + CpG
POPTR_0006s066902g	MBD; MeCP2, MBD1, MBD2, MBD3, MBD4, CLLD8-like and BAZ2A-like proteins constitute a family of proteins that share the methyl-CpG-binding domain (MBD)	promoter (1515bp)	Anderlingen	CpG
POPTR_0006s08960g	catalytic domain of protein kinases	promoter (1597 bp)	Wallstawe	CHG + CpG

POPTR_0006s09150g	aldo-keto reductases, a superfamily of soluble NAD(P)(H) oxidoreductases whose chief purpose is to reduce aldehydes and ketones to primary and secondary alcohols	gene body + promoter	Anderlingen	CHH + CHG
POPTR_0006s16120g	glutamate-1-semialdehyde 2,1-aminomutase	promoter (1230bp)	Wallstawe	CpG
POPTR_0006s18990g	member of cyclic nucleotide gated channel family	gene body + promoter	Anderlingen	CHH
POPTR_0006s20500g	elongation factor Tu family protein	gene body + promoter	Wallstawe	CHG
POPTR_0006s21230g	Vps51/Vps67 family (components of vesicular transport) protein	gene body	Wallstawe	CHH
POPTR_0006s22010g	diadenosine 5' family protein	promoter (62bp)	Wallstawe	CpG
POPTR_0006s22450g	Rossmann-like domain	gene body	Anderlingen	CpG
POPTR_0006s22680g	NAD(P)-binding domain	gene body + promoter	Anderlingen	CHG
POPTR_0006s25450g	Nup85 nucleoporin	gene body	Wallstawe	CpG
POPTR_0006s28950g	plant calmodulin-binding domain	gene body	Anderlingen	CHH
POPTR_0007s02230g	wall-associated receptor kinase galacturonan-binding	gene body	Wallstawe	CHG
POPTR_0007s02310g	catalytic domain of protein kinases	gene body	Wallstawe	CHG
POPTR_0007s02590g	secretory peroxidase and related secretory plant peroxidases	gene body	Anderlingen	CHH
POPTR_0007s02970g	exostosin	gene body	Anderlingen	CHH
POPTR_0007s03070g	putative glucosyltransferase family protein	gene body	Wallstawe	CHG
POPTR_0007s03470g	chromosome segregation protein SMC	promoter (1356bp)	Wallstawe	CpG
POPTR_0007s04120g	aspartyl protease family protein	promoter (1763bp)	Wallstawe	CpG
POPTR_0007s07470g	prefoldin alpha subunit, prefoldin is a hexameric molecular chaperone complex	promoter (879 bp)	Anderlingen	CHH
POPTR_0007s07590g	inorganic H+ pyrophosphatase	gene body	Anderlingen	CHH + CHG
POPTR_0007s07830g	histone-like transcription factor (CBF/NF-Y)	gene body	Wallstawe	CHH + CHG
POPTR_0007s11070g	red chlorophyll catabolite reductase	gene body	Wallstawe	CpG

POPTR_0008s01270g	allantoate amidohydrolase	gene body	Anderlingen	CpG
POPTR_0008s03700g	serine/threonine protein kinases	gene body	Anderlingen	CHH
POPTR_0008s08960g	ribosomal protein L32	gene body	Anderlingen	CHH
POPTR_0008s09320g	MPP superfamily, metallophosphatase superfamily	promoter (155 bp)	Anderlingen	CHH
POPTR_0008s13460g	flavin-binding kelch domain F-box family protein	gene body	Wallstawe	CpG
POPTR_0008s13710g	leucine-rich repeats (LRRs), ribonuclease inhibitor (RI)-like subfamily	gene body	Wallstawe	CHG
POPTR_0008s16650g	cryptochrome, plant family; DNA photolyase, FAD binding domain of DNA polymerase	gene body	Anderlingen	CpG
POPTR_0008s18310g	NBD sugar-kinase HSP70 actin superfamily	promoter (1875 bp)	Anderlingen	CHH
POPTR_0008s18840g	chromosome segregation protein SMC	gene body + promoter	Wallstawe	CHG
POPTR_0008s20220g	putative histidine-containing phosphotransfer protein 2	promoter (1732bp)	Anderlingen	CpG
POPTR_0008s21940g	zinc knuckle	gene body	Wallstawe	CHG
POPTR_0009s08380g	histidine phosphatase domain found in phosphoglycerate mutases and related proteins	gene body	Wallstawe	CpG
POPTR_0009s09810g	UDP-glucosyl transferase	gene body + promoter	Anderlingen	CHG
POPTR_0009s11510g	Cf-4/9 disease resistance-like family protein	promoter (1250 bp)	Anderlingen	CHH
POPTR_0009s15440g	retinal pigment epithelial membrane protein	gene body	Anderlingen	CHH
POPTR_0009s15600g	beta-glucosidase-related glycosidases	gene body	Anderlingen	CHH + CHG
POPTR_0009s17130g	lectin L-type, legume lectins	gene body + promoter	Wallstawe	CHH + CpG
POPTR_0010s04460g	actin cross-linking protein	promoter (1310bp)	Anderlingen	CpG
POPTR_0010s05110g	catalytic domain of protein kinases	gene body + promoter	Anderlingen	CHG
POPTR_0010s11680g	non-repetitive/WGA-negative nucleoporin family protein	gene body	Anderlingen	CHH
POPTR_0010s19000g	heavy-metal-associated domain (HMA)	gene body	Anderlingen	CHH
POPTR_0010s19570g	kinase family protein	gene body	Wallstawe	CHH
POPTR_0010s20390g	peptidases S8 3	gene body + promoter	Wallstawe	CHH
POPTR_0010s24430g	abhydrolase 6, alpha/beta hydrolase family	promoter (1555 bp)	Wallstawe	CHH

POPTR_0011s10635g	UBN2, gag-polypeptide of LTR copia-type	gene body	Anderlingen	CHH
POPTR_0011s10730g	PC-esterase; GDSL/SGNH-like acyl-esterase family	gene body	Wallstawe	CHG + CpG
POPTR_0011s14640g	peptidase family S64	gene body	Wallstawe	CHH + CHG
POPTR_0011s15760g	S-locus glycoprotein family	gene body + promoter	Wallstawe	CHH
POPTR_0011s15770g	galactose mutarotas 2, galactose mutarotase-like	gene body + promoter	Wallstawe	CHH
POPTR_0012s04860g	chaperonin family protein	gene body	Anderlingen	CpG
POPTR_0012s06310g	ubiquitin activating enzyme (E1) subunit APPBP1; APPBP1 is part of the heterodimeric activating enzyme (E1), specific for the Rub family of ubiquitin-like proteins	gene body	Anderlingen	CpG
POPTR_0012s06870g	DEAD-like helicases superfamily; diverse family of proteins involved in ATP-dependent RNA or DNA unwinding	promoter (387 bp)	Anderlingen	CpG
POPTR_0012s07680g	phosphatidylinositol 3-kinase family protein	promoter (1666 bp)	Wallstawe	CHG + CpG
POPTR_0012s12880g	C2 domain present in genes regulated by Cold 2 (SRC2)-like proteins	gene body	Anderlingen	CpG
POPTR_0012s13170g	PPR repeat family	promoter (1932bp)	Wallstawe	CpG
POPTR_0013s02480g	PRK06128; oxidoreductase	gene body	Anderlingen	CpG
POPTR_0013s07610g	PLATZ transcription factor	gene body	Anderlingen	CHH
POPTR_0013s10090g	rossmann-fold NAD(P)(+)-binding proteins	promoter (1675bp)	Wallstawe	CpG
POPTR_0013s10355g	zinc knuckle	gene body	Wallstawe	CHG
POPTR_0013s10710g	AAA+ (ATPases Associated with a wide variety of cellular activities) superfamily represents an ancient group of ATPases belonging to the ASCE (for additional strand, catalytic E) division of the P-loop NTPase fold	promoter (906 bp)	Anderlingen	CHH
POPTR_0013s12510g	6a-hydroxymaackiain methyltransferase family protein	gene body	Wallstawe	CHH
POPTR_0013s14910g	exocyst subunit EXO70 family protein	gene body	Anderlingen	CpG
POPTR_0014s00960g	NBS resistance protein	gene body	Wallstawe	CHG

POPTR_0014s01810g	peroxisomal membrane 22 kDa (Mpv17/PMP22) family protein	gene body + promoter	Anderlingen	CHH + CHG
POPTR_0014s01860g	FAD dependent oxidoreductase	promoter (842bp)	Anderlingen	CpG
POPTR_0014s02060g	cytochrome P450 family protein	gene body	Anderlingen	CHH + CpG
POPTR_0014s04140g	UDP-glucosyl transferase	gene body	Wallstawe	CpG
POPTR_0014s07040g	pre-50S ribosomal subunits, which implies a function in ribosome assembly	gene body	Wallstawe	CpG
POPTR_0014s15350g	cellulose synthase-like protein	gene body	Wallstawe	CHH
POPTR_0014s16310g	nucleoporin N; Nup133 N terminal like	gene body	Anderlingen	CHG
POPTR_0014s17340g	GT1-Gtf-like; family includes the Gtfs, a group of homologous glycosyltransferases	promoter (506bp)	Anderlingen	CpG
POPTR_0014s18950g	glycosyltransferase family A (GT-A) includes diverse families of glycosyl transferases with a common GT-A type structural fold	gene body	Wallstawe	CHG
POPTR_0014s18950g	glycosyltransferase like family	gene body + promoter	Wallstawe	CpG
POPTR_0015s00420g	F-box family protein	gene body	Wallstawe	CpG
POPTR_0015s07790g	RING-finger domain, a specialized type of Zn-finger of 40 to 60 residues that binds two atoms of zinc)	promoter (954bp)	Wallstawe	CpG
POPTR_0015s09330g	galactinol-sucrose galactosyltransferase	gene body + promoter; gene body	Anderlingen	CHH; CHG + CpG
POPTR_0015s10290g	protein kinase superfamily protein	promoter (675 bp)	Anderlingen	CHH
POPTR_0015s14510g	40 S ribosomal protein S20	promoter (713bp)	Anderlingen	CpG
POPTR_0015s15150g	leucine-rich repeats (LRRs), ribonuclease inhibitor (RI)-like subfamily	gene body	Wallstawe	CHG
POPTR_0016s00660g	STKc-phototropin-like; catalytic domain of phototropin-like protein serine/threonine kinases	gene body	Anderlingen	CpG
POPTR_0016s021501g	glycosyltransferase family 28 C-terminal domain	gene body + promoter	Wallstawe	CHG

POPTR_0016s04630g	cysteine/histidine-rich C1 domain family protein	gene body + promoter; gene body	Anderlingen; Wallstawe	CHH; CHH
POPTR_0016s04750g	cysteine/histidine-rich C1 domain family protein	gene body	Wallstawe	CpG
POPTR_0016s06380g	universal minicircle sequence binding protein	gene body	Anderlingen	CHH
POPTR_0016s07950g	chloroplast signal recognition particle subunit	gene body	Wallstawe	CpG
POPTR_0016s10360g	PRA1 family protein	promoter (1694 bp)	Anderlingen	CHG
POPTR_0016s11100g	putative GTPase activating protein for Arf	gene body	Anderlingen	CHG + CpG
POPTR_0016s11490g	zinc knuckle	gene body	Wallstawe	CHG
POPTR_0016s14620g	PQQ-dependent dehydrogenase, methanol/ethanol family	gene body; promoter (13 bp)	Anderlingen	CHH + CHG + CpG; CHH
POPTR_0017s00570g	NB-ARC domain-containing disease resistance protein	gene body	Wallstawe	CHH
POPTR_0017s00770g	leucine-rich repeats (LRRs)	gene body	Anderlingen	CpG
POPTR_0017s01260g	PTR2, POT family	gene body	Anderlingen	CHH
POPTR_0017s01300g	POT family	gene body	Anderlingen	CHH
POPTR_0017s02120g	zinc finger family protein	promtor (1944 bp)	Anderlingen	CHH
POPTR_0017s02540g	patatin-like phospholipase of plants	promoter (709 bp)	Wallstawe	CHG + CpG
POPTR_0017s03570g	transcription factor Tfb4	gene body + promoter	Anderlingen	CHG
POPTR_0017s04440g	leucine-rich repeat (LRR) receptor-like protein kinase	gene body + promoter	Wallstawe	CHH + CHG + CpG
POPTR_0017s04700g	CC-NBS-LRR resistance protein	gene body	Wallstawe	CHG + CpG
POPTR_0017s05000g	peptidase C48; Ulp1 protease family, C-terminal catalytic domain	gene body	Anderlingen	CHG
POPTR_0017s06300g	AAA+ (ATPases associated with a wide variety of cellular activities) superfamily represents an ancient group of ATPases belonging to the ASCE (for additional strand, catalytic E) division of the P-loop NTPase fold	gene body + promoter	Wallstawe	CHH
POPTR_0017s06960g	PPR repeat family	gene body	Wallstawe	CHH
POPTR_0017s09440g	PHA03247; large tegument protein UL36	gene body	Anderlingen	CHG
POPTR_0017s09660g	nodulin-like	gene body	Wallstawe	CHH

POPTR_0017s09830g	GYF domain: contains conserved Gly-Tyr-Phe residues	promoter (472 bp)	Wallstawe	CHG
POPTR_0017s10460g	SGNH hydrolase or GDSL hydrolase (diverse family of lipases and esterases)	promoter (1168 bp)	Anderlingen	CHG
POPTR_0017s13630g	nodule-enhanced malate dehydrogenase family protein	gene body	Anderlingen	CpG
POPTR_0017s137702g	DNA-dependent RNA polymerase	gene body	Anderlingen	CHH + CHG
POPTR_0017s14590g	T3/T7-like RNA polymerase	gene body	Anderlingen	CHH + CHG
POPTR_0018s04140g	trypsin-like peptidase domain	gene body	Anderlingen	CHH
POPTR_0018s11150g	tetratricopeptide repeat	gene body	Wallstawe	CpG
POPTR_0018s11370g	hydroxycinnamoyl-coenzyme A shikimate/quinate hydroxycinnamoyltransferase (HCT)	promoter (540 bp)	Anderlingen	CHH
POPTR_0018s11710g	omega-6 fatty acid desaturase	gene body	Wallstawe	CHH + CHG
POPTR_0018s14780g	vacuolar protein sorting-associated protein 26	promoter (1001 bp)	Anderlingen	CHH
POPTR_0019s00260g	leucine-rich repeat (LRR) receptor	gene body + promoter	Anderlingen	CHH
POPTR_0019s01690g	HXXXD-type acyl-transferase family protein	promoter (478 bp)	Anderlingen	CHH + CpG
POPTR_0019s01710g	Sec23/Sec24 protein transport family protein	promoter (1833 bp)	Anderlingen	CHH
POPTR_0019s02030g	leucine rich repeats (LRRs)	gene body	Anderlingen	CpG
POPTR_0019s06290g	tandem copies of the domain bind telomeric DNA tandem repeats as part of the capping complex	promoter (1336 bp)	Wallstawe	CHG + CpG
POPTR_0019s08830g	leucine-rich repeat receptor-like protein kinase	promoter (848bp)	Anderlingen	CpG
POPTR_0019s11980g	peptidase C1A subfamily	gene body	Wallstawe	CHH + CHG
POPTR_0019s14500g	exocyst subunit EXO70 family protein	gene body	Wallstawe	CpG
