# Supplementary material for: Site-Dependent Differences in DNA Methylation and Their Impact on Plant Establishment and Phosphorus Nutrition in Populus trichocarpa
Source: PLoS One. 2016 Dec 19;11(12):e0168623. doi: 10.1371/journal.pone.0168623 (PMC5167412; doi:10.1371/journal.pone.0168623)
Supplement: S4 Table — (PDF) [file pone.0168623.s012.pdf]

S4 Table. Description of differentially methylated regions (DMRs) occurring in annotated promoter and gene body sequences of the *Populus trichocarpa* genome.

DMR gene IDs	annotation	higher methylation site	^{m}C context
*POPTR*_0001s01660g	NBS-LRR resistance gene-like protein ARGH35	Wallstawe	CHG & CHH
*POPTR*_0001s06110g	dirigent-like protein, regulates coupling of monolignol plant phenols to generate the cell wall polymers lignins and lignans that are involved in structural fortification and defense against pathogens	Wallstawe	CHH
*POPTR*_0001s17820g	DNA polymerase III	Wallstawe	CHG
*POPTR*_0001s19460g	zinc knuckle – zinc ion binding, nucleic acid binding	Anderlingen	CHG
*POPTR*_0001s42010g	catalytic domain of protein kinases	Wallstawe	CHH
*POPTR*_0002s03260g	horseradish peroxidase and related secretory plant peroxidases	Anderlingen	CHG & CHH
*POPTR*_0002s07850g	component of the thylakoid-localized Sec system involved in the translocation of cytoplasmic proteins into plastid	Wallstawe	CHG
*POPTR*_0002s23200g	aconitate hydratase, encodes a aconitase that can catalyze the conversion of citrate to isocitrate through a cis-aconitate intermediate, indicating a role in the response to oxidative stress	Wallstawe	CHG
*POPTR*_0003s12640g	glycosyl hydrolase family 32, beta-fructosidases	Wallstawe	CHH
*POPTR*_0005s01450g	PPR repeat family, DYW family of nucleic acid deaminases	Wallstawe	CHH
*POPTR*_0005s08440g	NAC domain protein	Wallstawe	CHG
*POPTR*_0005s11730g	AAA+ superfamily represents an ancient group of ATPases belonging to the ASCE division of the P-loop NTPase fold	Anderlingen	CHH
*POPTR*_0006s02510g	glutathione S-transferases	Anderlingen	CHH

DMR gene IDs	annotation	higher methylation site	^{m}C context
*POPTR*_0006s09150g	aldo-keto reductases, a superfamily of soluble NAD(P)(H) oxidoreductases whose chief purpose is to reduce aldehydes and ketones to primary and secondary alcohols	Anderlingen	CHG & CHH
*POPTR*_0006s18990g	effector domain of the CAP family of transcription factors, members include CAP (or cAMP receptor protein (CRP)), which binds cAMP, FNR (fumarate and nitrate reduction)	Anderlingen	CHH
*POPTR*_0006s20500g	elongation factor Tu family protein	Wallstawe	CHG
*POPTR*_0006s22680g	NAD(P)-binding Rossmann-like domain	Anderlingen	CHG
*POPTR*_0008s18840g	chromosome segregation protein	Wallstawe	CHG
*POPTR*_0009s09810g	UDP-glucosyl transferase	Anderlingen	CHG
*POPTR*_0009s17130g	lectin L-type, legume lectins	Wallstawe	CpG & CHH
*POPTR*_0010s05110g	catalytic domain of protein kinases	Anderlingen	CHG
*POPTR*_0010s20390g	peptidases S8 3	Wallstawe	CHH
*POPTR*_0011s15760g	S-locus glycoprotein family	Wallstawe	CHH
*POPTR*_0011s15770g	galactose mutarotase-like	Wallstawe	CHH
*POPTR*_0014s01810g	peroxisomal membrane 22 kDa family protein	Anderlingen	CHG & CHH
*POPTR*_0014s18950g	glycosyltransferase like family	Wallstawe	CpG
*POPTR*_0015s09330g	galactinol-sucrose galactosyltransferase	Anderlingen	CHH
*POPTR*_0016s021501g	glycosyltransferase family 28 C-terminal domain	Wallstawe	CHG
*POPTR*_0016s04630g	cysteine/histidine-rich C1 domain family protein	Anderlingen	CHH
*POPTR*_0017s03570g	transcription factor Tfb4, TFIIH (subunit of the general trancriptions factors for promotor recognition and initiation of transcription)	Anderlingen	CHG
*POPTR*_0017s04440g	leucine-rich repeat receptor-like protein kinase	Wallstawe	CHG & CHH & CpG
*POPTR*_0017s06300g	AAA+ superfamily represents an ancient group of ATPases belonging to the ASCE division of the P-loop NTPase fold	Wallstawe	CHH
*POPTR*_0019s00260g	LRR 8, leucine rich repeat	Anderlingen	CHH
