# Supplementary material for: Site-Dependent Differences in DNA Methylation and Their Impact on Plant Establishment and Phosphorus Nutrition in Populus trichocarpa
Source: PLoS One. 2016 Dec 19;11(12):e0168623. doi: 10.1371/journal.pone.0168623 (PMC5167412; doi:10.1371/journal.pone.0168623)
Supplement: S5 Table — (PDF) [file pone.0168623.s013.pdf]

S5 Table. Loci of differential DNA methylation in coding regions of clonal *Populus trichocarpa* derived from two short rotation forestry sites (Anderlingen vs. Wallstawe).

differentially methylated gene ID	annotation	differentially methylated coding region	higher methylation level site	cytosine context
*POPTR*_0008s20220	putative histidine-containing phosphotransfer protein 2	promoter	Anderlingen	CpG
*POPTR*_0010s11680	non-repetitive/WGA-negative nucleoporin family protein	gene body	Anderlingen	CHH
*POPTR*_0012s04860	chaperonin family protein	gene body	Anderlingen	CpG
*POPTR*_0017s02120	zinc finger family protein	promoter	Anderlingen	CHH
*POPTR*_0017s14590	T3/T7-like RNA polymerase	gene body	Anderlingen	CHG & CHH
*POPTR*_0018s14780	vacuolar protein sorting-associated protein 26	promoter	Anderlingen	CHH
