# Supplementary material for: Site-Dependent Differences in DNA Methylation and Their Impact on Plant Establishment and Phosphorus Nutrition in Populus trichocarpa
Source: PLoS One. 2016 Dec 19;11(12):e0168623. doi: 10.1371/journal.pone.0168623 (PMC5167412; doi:10.1371/journal.pone.0168623)
Supplement: S6 Table — (PDF) [file pone.0168623.s014.pdf]

**S6 Table. Pearson's product-moment correlation of methylation state in differentially methylated genes and gene expression in plant material from clonal *Populus trichocarpa* (cv. Muhle Larson) cuttings derived from two different short rotation forestry sites (Anderlingen vs. Wallstawe).**

| sites | tissue | treatment | correlation coefficient (r) | p-value |
|---|---|---|---|---|
| Anderlingen | | +P | -0.57 | 0.0522 |
| | leaves | -P | -0.50 | 0.3156 |
| | | +P | -0.39 | 0.2150 |
| | roots | -P | -0.75 | 0.0862 |
| Wallstawe | | +P | -0.80 | 0.0018 |
| | leaves | -P | -0.52 | 0.2860 |
| | | +P | -0.71 | 0.0100 |
| | roots | -P | -0.87 | 0.0227 |
