# Supplementary material for: Site-Dependent Differences in DNA Methylation and Their Impact on Plant Establishment and Phosphorus Nutrition in Populus trichocarpa
Source: PLoS One. 2016 Dec 19;11(12):e0168623. doi: 10.1371/journal.pone.0168623 (PMC5167412; doi:10.1371/journal.pone.0168623)
Supplement: S7 Table — Here, the maximum expectation value scores the complementarity between small RNA and their target transcript. With a lower maximum expectation value (0–2.0), a more stringent cut-off threshold and thereby a lower false positive prediction is set. (PDF) [file pone.0168623.s015.pdf]

S7 Table. Description of possible genes targeted by differentially methylated miRNAs in *Populus trichocarpa*.

miRNA	target gene	expression in leaves or roots	maximum expectation[1]
Ptc-miR1446a-e	*POPTR*_0013s14900g	Yes	1.5
Ptc-miR1446a-e	*POPTR*_0006s04360g	Yes	2.0
Ptc-miR481ab	*POPTR*_0012s01480g	No	1.5
Ptc-miR481cd	*POPTR*_0013s11260g	No	1.5
Ptc-miR6432	*POPTR*_0004s02320g	Yes	2.0
Ptc-miR827	*POPTR*_0006s09220g	Yes	2.0
